# Shared processes resolve competition within and between episodic and semantic memory: Evidence from patients with LIFG lesions

**DOI:** 10.1016/j.cortex.2018.07.007

**Published:** 2018-11

**Authors:** Sara Stampacchia, Hannah E. Thompson, Emily Ball, Upasana Nathaniel, Glyn Hallam, Jonathan Smallwood, Matthew A. Lambon Ralph, Elizabeth Jefferies

**Affiliations:** a Department of Psychology, University of York, UK; b School of Psychology, University of Surrey, UK; c School of Human and Health Sciences, University of Huddersfield, UK; d Neuroscience and Aphasia Research Unit (NARU), Division of Neuroscience & Experimental Psychology, School of Biological Sciences, University of Manchester, UK; e Department of Psychology, University of Haifa, Israel

**Keywords:** Episodic memory, Semantic memory, Controlled retrieval, LIFG, Stroke aphasia

## Abstract

Semantic cognition is supported by two interactive components: semantic representations and mechanisms that regulate retrieval (cf. ‘semantic control’). Neuropsychological studies have revealed a clear dissociation between semantic and episodic memory. This study explores if the same dissociation holds for control processes that act on episodic and semantic memory, or whether both types of long-term memory are supported by the same executive mechanisms. We addressed this question in a case-series of semantic aphasic patients who had difficulty retrieving both verbal and non-verbal conceptual information in an appropriate fashion following infarcts to left inferior frontal gyrus (LIFG). We observed parallel deficits in semantic and episodic memory: (i) the patients' difficulties extended beyond verbal materials to include picture tasks in both domains; (ii) both types of retrieval benefitted from cues designed to reduce the need for internal constraint; (iii) there was little impairment of both semantic and episodic tasks when control demands were minimised; (iv) there were similar effects of distractors across tasks. Episodic retrieval was highly susceptible to false memories elicited by semantically-related distractors, and confidence was inappropriately high in these circumstances. Semantic judgements were also prone to contamination from recent events. These findings demonstrate that patients with deregulated semantic cognition have comparable deficits in episodic retrieval. The results are consistent with a role for LIFG in resolving competition within both episodic and semantic memory, and also in biasing cognition towards task-relevant memory stores when episodic and semantic representations do not promote the same response.

## 1 Introduction

Neuropsychological studies provide compelling evidence for the existence of separable episodic and semantic memory stores. Patients with semantic dementia have progressive yet selective degeneration of conceptual knowledge across all tasks and input modalities, which correlates with the degree of atrophy in the anterior ventrolateral temporal lobes (Butler et al., 2009, Mummery et al., 2000), yet their memory for recent episodic events is largely intact (Graham and Hodges, 1997, Graham et al., 1997, Graham et al., 2003, Graham et al., 2000). In contrast, anterograde amnesia is characterised by poor encoding and retrieval of specific events as opposed to factual information, following damage to the hippocampus and associated structures in the medial temporal lobes (Nadel and Moscovitch, 1997, Nestor et al., 2006, Vargha-Khadem et al., 1997). These findings suggest that anterior ventrolateral temporal cortex supports conceptual generalisation across experiences, while hippocampus promotes pattern separation for recently-encoded episodes (Kumaran and McClelland, 2012, McClelland et al., 1995).

Studies also point to the existence of contrastive types of semantic deficit. The term “semantic aphasia” was first coined by Head (1926) to describe patients showing difficulties in shaping and manipulating knowledge to serve symbolic processing – in the presence of heterogenous language impairments – rather than loss of semantic knowledge per se. In line with Head's clinical description, studies have shown that, unlike the degraded knowledge in semantic dementia, patients with semantic aphasia (SA) show deregulated semantic cognition across different tasks and input modalities following left frontoparietal stroke (Jefferies and Lambon Ralph, 2006, Jefferies et al., 2008, Rogers et al., 2015). SA patients show inconsistent semantic performance when the same concepts are tested under different control demands, as well as sensitivity to cues and miscues that constrain retrieval or increase the availability of irrelevant knowledge (Corbett et al., 2011, Jefferies et al., 2008, Noonan et al., 2010). They have difficulty retrieving non-dominant aspects of knowledge and dealing with competition from strong yet irrelevant semantic distractors during semantic retrieval (Almaghyuli et al., 2012, Noonan et al., 2010). These problems extend beyond language, to affect sound, picture and action understanding (Corbett et al., 2009a, Corbett et al., 2009b, Corbett et al., 2011, Gardner et al., 2012, Thompson et al., 2015). Collectively this evidence shows that SA patients have multimodal deficits of semantic control, i.e., they find it difficult to flexibly retrieve and shape semantic knowledge to suit the task or circumstances and show impairment when there is a need to resolve competition between different meanings or features of concepts. The distinction between semantic dementia and patients with SA supports a component process account, in which semantic cognition emerges from interactions between transmodal conceptual representations and control processes (Controlled Semantic Cognition Framework; Jefferies, 2013, Lambon Ralph et al., 2017).

This proposal is also pertinent to understanding differences in episodic memory deficits in amnesia (see Blumenfeld & Ranganath, 2007 for a review). In contrast to patients with circumscribed medial temporal lobe injury (such as HM, Scoville & Milner, 1957), patients with additional prefrontal involvement show better cued than free recall (Mangels et al., 1996, della Rocchetta and Milner, 1993) and disproportionate difficulty in retrieving word-pairs previously associated with other targets, reflecting a failure to overcome proactive interference (Shimamura, Jurica, Mangels, Gershberg, & Knight, 1995). In both semantic and episodic tasks, bringing to mind unusual associations, or task-relevant knowledge in the face of strong competition, might involve promoting specific aspects representations and suppressing irrelevant dominant information (Anderson, 1988; Badre and Wagner, 2007, Whitney et al., 2011). The similarity of these theoretical accounts fuels interest in whether they have a shared or distinct neural basis.

**Fig. 1:**
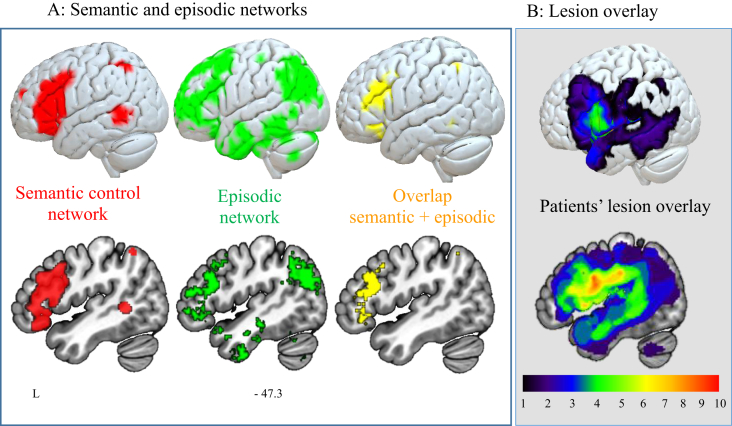
Brain networks implicated in semantic and episodic retrieval overlap with patients' lesions. (A) Semantic control network (red, from Noonan et al., 2013, adapted by Humphreys and Lambon Ralph, 2015), episodic memory network (green, from Neurosynth; a meta-analysis of 393 studies containing the term “episodic”), the overlap of the two networks (yellow). Rendered views are displayed using Surfice (https://www.nitrc.org/projects/surfice/); sagittal views using MRIcroGL (http://www.cabiatl.com/mricrogl/). The overlap mask included only one cluster of a minimum of 50 voxels which corresponded to mid-to-post LIFG, pars triangularis extending to pars opercularis and middle frontal gyrus (MNI -48, 24, 24). (B) Lesion overlay of the sample of SA patients included in the study. Patients' brains compared to aged-matched controls. Grey matter, white matter and CSF were segmented and changes from the healthy control brains were highlighted as ‘lesion’ using automated methods (Seghier et al., 2008). Colour bar indicates amount of overlap from 1 to 10 patients.

Functional neuroimaging studies suggest that overlapping networks are important for the control of episodic and semantic memory (see Fig. 1A). Left inferior frontal gyrus (LIFG) has a well-established role in the control of episodic memory: it shows a stronger response in the retrieval of weakly vs. strongly-encoded memories (Barredo et al., 2015, Hayes et al., 2011) and is engaged by interference resolution (Badre and Wagner, 2005, Wimber et al., 2009). Likewise, this region shows increased activation in semantic retrieval for ambiguous words, weak associations or strong distracters (for a meta-analysis, see Noonan, Jefferies, Visser, & Lambon Ralph, 2013; also Badre and Wagner, 2005, Badre and Wagner, 2007, Thompson-Schill et al., 1997). Controlled retrieval from episodic and semantic memory partially overlaps with “multiple-demand regions” that are engaged for difficult tasks across multiple domains; however, anterior LIFG lies outside this network and appears to specifically support the control of memory (Badre et al., 2005, Davey et al., 2016, Nelson et al., 2009). In line with this proposal, inhibitory transcranial magnetic stimulation to LIFG disrupts control-demanding semantic judgements but not more automatic aspects of semantic retrieval or demanding non-semantic judgements (Gough et al., 2005, Hallam et al., 2016, Hoffman et al., 2010, Krieger-Redwood and Jefferies, 2014, Whitney et al., 2011).

Despite these similarities, few studies have directly compared manipulations of difficulty across episodic and semantic judgements. It is unclear whether LIFG contributes to episodic memory indirectly by regulating conceptual retrieval or whether LIFG is crucial for regulating retrieval from both memory stores. Neuropsychology can help to resolve this theoretical uncertainty by establishing if damage to LIFG gives rise to symmetrical deficits of episodic and semantic memory. Semantic and episodic representations often mutually support retrieval: to understand the semantic link between items like dog and beach, we can bring to mind specific episodes in which these items co-occurred (Westmacott and Moscovitch, 2003, Westmacott et al., 2004). Similarly, in event memory, we draw on semantic representations of related episodes to support encoding and retrieval, giving rise to “levels of processing effects” (Anderson, 1981, DeWitt et al., 2012). We therefore need the capacity to select a response from one or other system, depending on the task demands. The inappropriate application of semantic information in an episodic context can give rise to false memories (Roediger, Balota, & Watson, 2001; Roediger & McDermott, 1995) and the engagement of LIFG might help to avoid these errors (Dennis et al., 2014, Garoff-Eaton et al., 2007, Kim and Cabeza, 2007).

In this study, we examined chronic post-stroke patients with SA and well-documented deficits of semantic control following LIFG lesions. To date, there has been little research on episodic memory in aphasia, including SA. We therefore investigated whether SA patients would show episodic deficits resembling their semantic impairment – namely, multimodal difficulties across verbal and non-verbal tasks, and sensitivity to cues that reduce the requirement for internally-constrained retrieval. We assessed whether semantic control impairment would elicit ‘false episodic memories’. In addition, to establish if semantic deficits directly underpin poor episodic memory or, alternatively, whether LIFG is critical for memory control across domains, we considered whether LIFG lesions would elicit ‘false semantic associations’ when semantic retrieval is preceded by task-irrelevant episodic encoding. Patients with multimodal semantic deficits following infarcts within LIFG may have difficulty resolving competition between episodic and semantic memory and their responses might reflect task-irrelevant memory representations, if LIFG plays a general role in regulating retrieval from both systems.

## 2 Participants

### 2.1 Patients

The study was approved by the local ethical committee and informed consent was obtained. Ten participants [six females; M (SD): Age = 62.8 (11.2); Age left education: 16.4 (1.2); years since CVA: 8.9 (5.6)] with chronic stroke aphasia from a left-hemisphere CVA were recruited from communication groups in Yorkshire, UK. Demographic details are provided in Supplementary Table 1. On the basis of their aphasic symptomatology they could be classified as follows: two Global; two Mixed Transcortical; five Transcortical Sensory/Anomic; one Broca. In line with the inclusion criteria adopted by Jefferies and Lambon Ralph (2006), patients were selected to show difficulties accessing semantic knowledge in both verbal and non-verbal tasks.

We previously found that such multimodal semantic deficits in stroke aphasia reflect difficulties with controlled access to semantic information (Corbett et al., 2009a, Corbett et al., 2011, Gardner et al., 2012, Noonan et al., 2010, Thompson et al., 2015), and this pattern was reproduced in this sample (see Background Neuropsychological Testing). All the patients showed greater difficulty on semantic tasks when control demands were high. In line with our previous results, we expected patients to show (i) a strong influence of word ambiguity, with poorer performance for subordinate meanings (assessed using the Ambiguity task below); (ii) strong effects of cueing and miscuing (in the Ambiguity task); (iii) poor inhibition of strong competitors (assessed using the Synonym judgment task with distractors); (iv) difficulty accessing non-canonical functions and uses of objects (assessed using the Object Use task). We also expected inconsistent performance – at the group level – on semantic tasks probing the same concepts with different control demands (assessed using the Cambridge semantic battery).

### 2.2 Lesion analysis

We used an automated method for identifying lesioned tissue: grey matter, white matter and CSF were segmented and changes from the healthy control brains were highlighted as ‘lesion’ (Seghier, Ramlackhansingh, Crinion, Leff, & Price, 2008). A lesion map generated using this approach is shown in Fig. 1. In addition, we manually assessed lesions of individual patients by tracing MRI scans onto standardized templates (Damasio & Damasio, 1989). All ten patients had lesions affecting posterior LIFG (see Fig. 1B and Supplementary Table 2); in seven cases, this damage extended to mid-to-anterior LIFG. Some lesions extended to inferior parietal and/or posterior temporal regions, with less overlap between cases in these additional regions. Three patients (P1, P3, P7) showed some degree of damage in the ATL. However, ventral ATL, which has been implicated in conceptual representation across modalities (Binney et al., 2012, Visser et al., 2012), was intact in all ten cases. This region is supplied by both the anterior temporal cortical artery of the middle cerebral artery and the anterior temporal branch of the distal posterior cerebral artery, reducing its vulnerability to stroke (Borden, 2006, Conn, 2008, Phan et al., 2005). The hippocampus was also intact. Fig. 1B provides a lesion overlay for the patient group, showing common lesions in regions of LIFG implicated in semantic control and episodic retrieval in neuroimaging studies of healthy participants.

### 2.3 Controls

Performance was compared for patients and healthy controls (N = 10 to 15, across different studies). None of the controls had a history of psychiatric or neurological disorder. They were matched to the patients on age and years of education (*p* > .06 across all comparisons).

## 3 Background neuropsychological testing

### 3.1 Non-semantic tests

Data for individual patients is shown in Supplementary Table 4. The “cookie theft” picture description (Goodglass & Kaplan, 1983) revealed non-fluent speech in half of the patients. Word repetition (PALPA 9; Kay, Lesser, & Coltheart, 1992) was also impaired in five patients out of ten. Executive/attentional impairment was seen in seven of the ten patients (see Supplementary Table 4), across four tasks: Elevator Counting with and without distraction from the Test of Everyday Attention (Robertson, Ward, Ridgeway, & Nimmo-Smith, 1994); Ravens Coloured Progressive Matrices (RCPM; Raven, 1962); Brixton Spatial Rule Attainment task (Burgess & Shallice, 1997) and Trail Making Test A & B (Reitan, 1958). This is in line with previous studies which found that deregulated semantic cognition correlated with executive dysfunction in stroke aphasia (Jefferies and Lambon Ralph, 2006, Noonan et al., 2010). Digit span was impaired in all patients, while 7 out of 10 had spatial spans in the normal range. The patients showed normal performance in the Face Recognition task from the Wechsler Memory Scale (WMS-III, Wechsler, 1997), which has minimal control demands. This confirms they were not amnesic. In contrast, the Verbal Paired Associates test from WMS-III was impaired (see below).

### 3.2 Cambridge semantic battery

This assesses semantic retrieval for a set of 64 items across tasks (Adlam et al., 2010, Bozeat et al., 2000), including picture naming, word-picture matching, verbal and pictorial semantic associations (Camel and Cactus Test, CCT). In line with their varying language output impairment, patients showed large variability during picture naming [percentage correct M (SD) = 63.3 (37.6)]. In contrast, performance was uniformly at ceiling in word-picture matching [M (SD) = 95.9 (5.5)]. When secondary associations between concepts were to be retrieved on the CCT – i.e., control demands were higher – performance was lower with no differences across modalities [words M (SD) = 78.3 (16.3); pictures M (SD) = 77.7 (13.6)]. Individual test scores are provided in Supplementary Table 3. Pairwise correlations between the six combinations of these four tasks revealed a correlation across word and picture association judgements [r = .63, *p* = .05]. The word and picture trials were probing the same association and therefore had highly correlated control demands. All other pairwise correlations were not significant [*p* ≥ .08]. This replicates the findings of Jefferies and Lambon Ralph (2006), who showed correlations across modalities within the same task (when control demands remained constant) but not between tasks with different controlled retrieval requirements.

### 3.3 Tests of semantic control

**Fig. 2:**
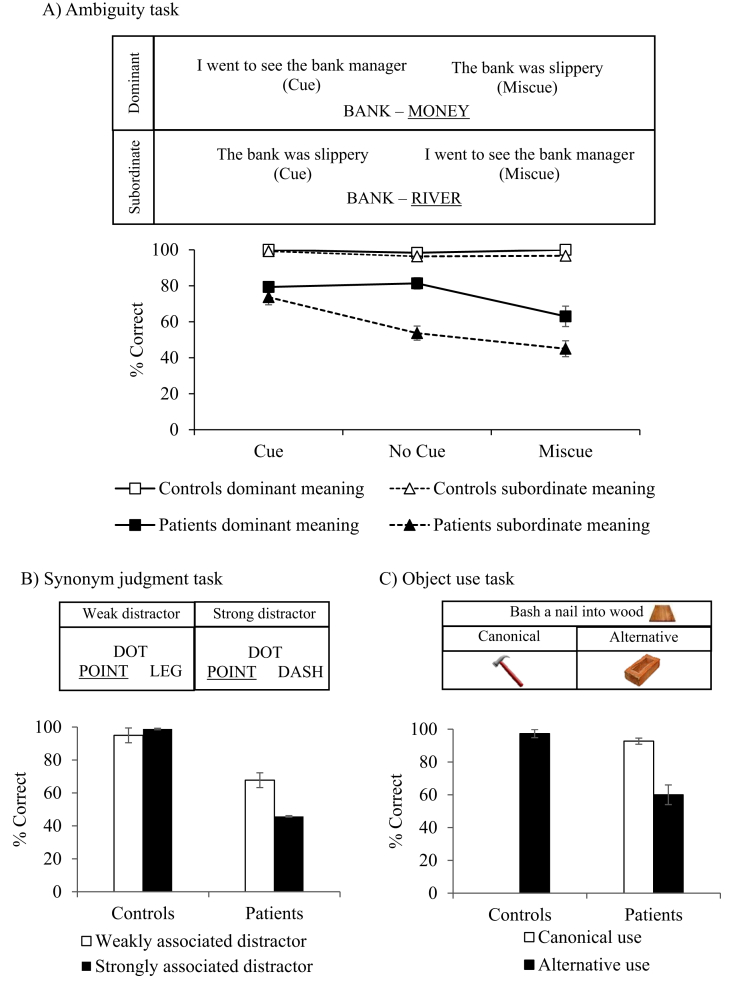
Tests manipulating semantic control. (A) Ambiguity task, from Noonan et al. (2010). (B) Synonym judgement task, from Samson et al. (2007). (C): Object use task, from Corbett et al. (2011). Error bars show SE of mean.

In line with the original use of the term “semantic aphasia” by Henry Head (1926) and the findings of Jefferies and Lambon Ralph (2006), the patients in this study had deficits affecting the appropriate use of concepts presented as words and objects. We presented three tasks that manipulated the control demands of verbal and non-verbal semantic judgements. See Fig. 2 for task descriptions and group-level results and Supplementary Table 3 for individual data.

#### 3.3.1 Ambiguity task

Semantic judgements (60 items) probed the dominant (money) and subordinate (river) meanings of ambiguous words (e.g., bank). These semantic decisions were preceded by no cue, or by a sentence that primed the relevant meaning (cue condition e.g., for money, i went to see the bank manager) or irrelevant interpretation (miscue condition e.g., the bank was slippery; Fig. 2A), from Noonan et al., 2010. There were four response options on each trial. All the patients were below the normal cut-off in all conditions. Every individual patient showed better comprehension for dominant than for subordinate interpretations [no cue condition percentage correct: dominant M (SD): 81.3 (9.9); subordinate M (SD) = 53.7 (12.4)]. In addition, every single patient showed additional impairment in accessing subordinate meaning following miscues rather than cues [percentage correct subordinate trials: miscues M (SD) = 45.0 (14.0); cues M (SD) = 73.7 (13.4)]. Patients’ performance was compared against controls using ANOVA, including dominance (dominant; subordinate), cueing (miscue; no cue; cue) and group (SA patients vs. controls). There were main effects of dominance [F(1,16) = 86.23, *p* < .001] and cueing [F(2,15) = 17.38, *p* < .001] plus interactions of dominance by cueing [F(2,15) = 8.34, *p* = .004], dominance by group [F(1,16) = 52.86, *p* = .001], cueing by group [F(2,15) = 14.81, *p* < .001] and the three-way interaction [F(2,15) = 6.00, *p* = .012; control data from Noonan et al., 2010; Fig. 2A].

#### 3.3.2 Synonym judgment task

We tested synonym judgement with strong or weak distractors (84 trials), from Samson, Connolly, & Humphreys, 2007; e.g., dot with point [target], presented with dash [strong distractor] or leg [weak distractor; Fig. 2B]. There were three response options per trial. Accuracy was below the cut-off for all patients and poorer when semantically-related but irrelevant distractors were presented [percentage correct: weak M (SD): 67.7 (11.4); strong M (SD): 45.8 (13.5)]. Patients’ performance was compared against controls using a 2 by 2 mixed ANOVA [main effect of condition: F(1,15) = 10.19, *p* = .006; group interaction: F(1,15) = 20.81, *p* < .001; Fig. 2B; control data from Samson et al., 2007].

#### 3.3.3 Object use task

The object use task (74 items), from Corbett et al. (2011), involved selecting an object to accomplish a task (e.g., bash a nail into wood), with all items represented as photographs. The target was either a canonical tool, normally used to complete the task (e.g., hammer), or an alternative non-canonical option (e.g., brick), presented among a set of five unsuitable distractors. All patients were poorer at selecting non-canonical than canonical targets [percentage correct: canonical M (SD) = 92.7 (7.9); alternative M (SD) = 60 (19); t(9) = 8.34, *p* < .001] and almost all were impaired compared to controls [t(16) = −5.47, *p* < .001, see Fig. 2C; control data from Corbett et al., 2011 and not collected for the canonical condition given near-ceiling performance]. One single patient (P5) was not below the normal cut-off in the non-canonical condition, however this patient was impaired at the pictorial version of the CCT.

The SA group showed strong sensitivity to all these control manipulations (Fig. 2) – i.e., more impaired comprehension of subordinate than dominant interpretations of ambiguous words; sensitivity to cues and miscues; better comprehension with weak than strong distractors and better retrieval of canonical than alternative object use. A composite score reflecting each patient's deficits in semantic cognition was derived from the Camel and Cactus Test and the three semantic control tasks described above using factor analysis. Patients are ordered by this composite score in the graphs and tables below.

In the next section, we examined whether our participants with deregulated semantic retrieval would show parallel deficits of episodic memory, including benefits of cues designed to constrain retrieval in both domains.

## 4 Verbal paired associate recall with cueing

### 4.1 Method

In a Verbal Paired Associates task (WMS-III, Wechsler, 1997), participants learned eight pairs of unrelated words (e.g., bank-cartoon). These were presented aurally four times, in a different order each time. Participants then attempted to recall the associate aloud from the probe. When there was no correct response, participants were given progressive phonological cues (i.e., the target's initial phonemes, one at a time) to reduce the need for internal constraints on episodic recall, e.g., “c.. ca.. car.. cart.. cartoo..”. Progressive phonological cues have already been shown to benefit semantic retrieval in SA (Jefferies et al., 2008, Noonan et al., 2010, Soni et al., 2009). The task was administered to eight patients; two with poor speech production were not tested (P1 and P7).

### 4.2 Results

#### 4.2.1 Accuracy

**Fig. 3:**
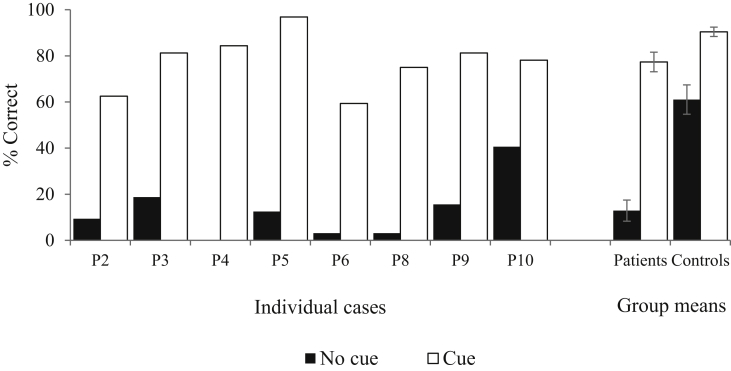
Verbal paired associate recall with phonological cueing (adapted from WMS-III, Wechsler, 1997). Error bars show SE of mean.

In the no-cue condition, patients’ accuracy was significantly lower than controls [t(21) = 5.12; *p* < .001]. Both patients and controls benefited from phonemic cueing [F(1,21) = 148.87, *p* < .001], but patients showed a stronger cueing effect than controls [cueing by group interaction: F(1,21) = 20.81, *p* < .001; Fig. 3]. In an individual analysis, every patient showed a significant improvement in performance after cueing [McNemar *p* < .001].

#### 4.2.2 Error analysis

Errors in the no cue condition were assigned to one of five categories: semantically-related to probe/target; interference (probe or target from a different pair); perseveration (repeating an inaccurate response given on a previous trial); phonologically-related to probe (sharing at least one phoneme in the correct position); unrelated. Omissions were disregarded. Four patients (P2 = 62%, P3 = 25%, P4 = 43%, P6 = 24%) produced semantically-related words in response to the probe (e.g., star-ladder → “star-heaven”; elephant-glass – “elephant-giraffe”). There were insufficient numbers of errors for statistical analysis, especially amongst control participants (although this pattern is explored in alternative-forced-choice recognition tasks below).

## 5 Paired associate recognition tasks

### 5.1 Rationale

As some patients had impaired speech production, the experiments below examined recognition. Experiment 1 manipulated the semantic relatedness of the probe and target words, the strength of episodic encoding, and the presence or absence of semantic distractors designed to elicit false episodic memories. Experiment 2 followed a similar structure but all of the words were semantically unrelated, to establish if episodic recognition was impaired relative to controls even when the role of meaning in encoding and retrieval was minimised. Experiment 3 presented pictures, not words, to establish if the multimodal nature of the semantic deficit would extend to episodic memory. We also asked participants to rate how confident they were in each decision on a scale from one (not confident at all) to seven (very confident).

### 5.2 Method

#### 5.2.1 Experiment 1

**Fig. 4:**
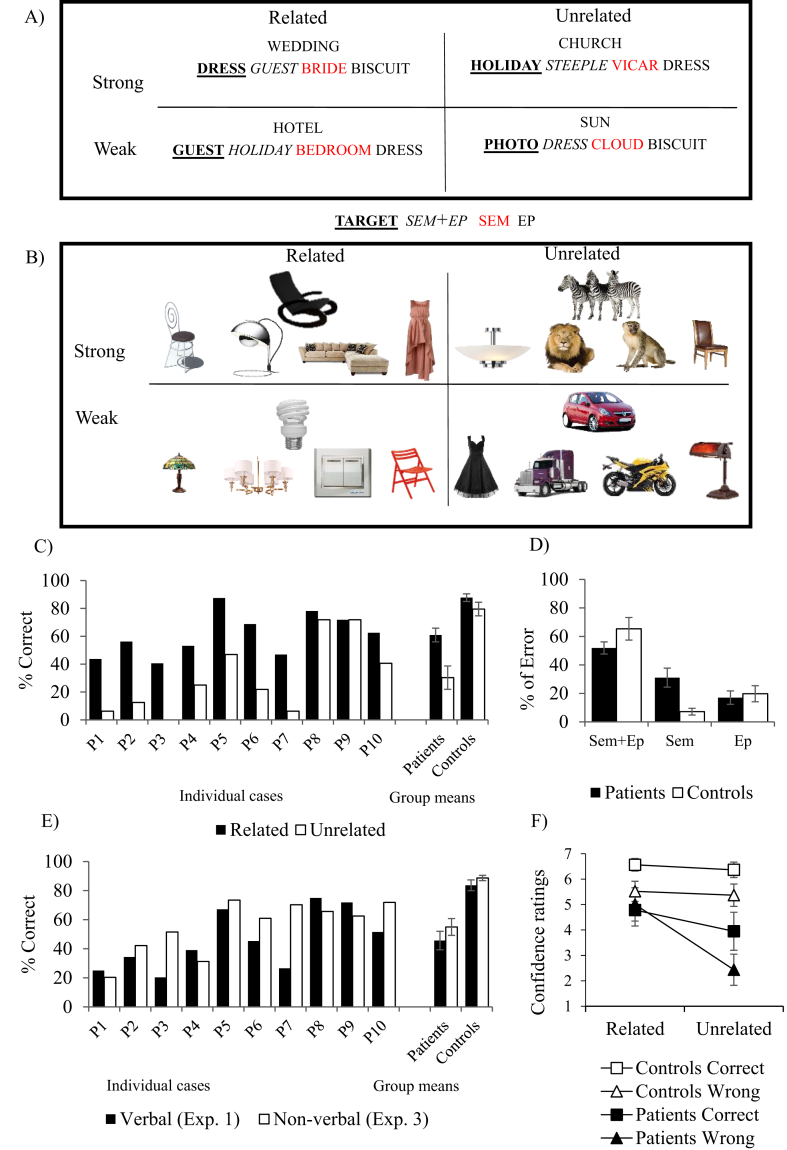
Paired associate recognition tasks and key results. A) Experiment 1 (words). B) Experiment 3 (pictures). Related and Unrelated conditions: probe paired with a semantically related or unrelated target at encoding. Strong trials: repeated 5 times at encoding; Weak trials: presented only once at encoding. Response options: Target – item paired with the probe at encoding; SEM distractor – novel and semantically related to the probe; SEM + EP distractor – semantically related to the probe and a target word for another probe; EP distactor – target on a different trial but not semantically related to the probe. Response options are displayed in the same order in both tasks. C) Effect of relatedness on accuracy in Experiment 1; D) Errors in Experiment 1; E) Modality effect: Experiment 1 vs. 3. F) Confidence analysis for Experiment 1: relatedness by accuracy by group. Error bars show SE of mean.

Participants tried to remember which two words were presented together as a pair. There were two manipulations during the learning phase, relatedness and episodic strength. Word-pairs were either semantically related or unrelated; they were also repeated five times or only once (see Fig. 4A and Appendix Table 1 for list of stimuli). Each probe word was paired with both a related and an unrelated target in separate lists, allowing us to examine interference errors. Latent Semantic Analysis (LSA; Laham & Steinhart, 1998) established stronger associations for related vs. unrelated trials [Related M (SD) = .32 (.15) vs. Unrelated M (SD) = .09 (.08); t(31) = 8.02, *p* < .001]. There were no LSA differences between other conditions [t(15) < 1].

In each encoding block, eight word-pairs were presented consecutively on a screen using E-Prime 2.0. Probes and targets were initially presented individually for 1000 msec and then the word-pair appeared on the screen for 3000 msec. The words were read aloud by the researcher. Immediately after encoding, participants performed a recognition task in which they were asked to select the word previously presented with the probe, from amongst four response options. On each trial, there was a novel semantic distractor related to the probe (SEM); an episodic distractor that was a target on a different trial (EP); and a semantic-episodic distractor that was both semantically related to the probe and a target for another probe (SEM + EP). LSA showed that semantically-related distractors were more associated to the probe than episodic distractors [SEM vs. EP: t(30) = 7.80, *p* < .001; SEM + EP vs. EP: t(63) = 10.28, *p* = .001]. The targets and different distractor types were matched for frequency, length and imageability [t < 1, *p* > .31]. Patients indicated their choice by pointing. The order of recognition trials was randomised for each participant. There were 8 word pairs per learning list, and 8 lists presented in a counterbalanced order across participants, providing 64 trials for analysis. To ensure that patients comprehended the instructions, the task was preceded by practice trials testing memory for four words pairs. When the response was wrong, the correct answer was provided, and the practice procedure was repeated until the participant showed complete understanding. In Experiments 2 and 3 this was not necessary since patients were already familiar with the task. Patients’ showed insight about their accuracy in all three experiments (see confidence analysis in section 5.3.5), confirming understanding of task instructions.

#### 5.2.2 Experiment 2

In a subsequent experiment, we used the same task structure but eliminated semantic links between the stimuli, using LSA scores of .5 or below [See Appendix Table 2 for list of stimuli]. Targets and distractors were matched to the items presented in Experiment 1 for frequency (using CELEX, Max Planck Institute for Psycholinguistics, 2001) and letter length [t ≤ 1.14, *p* ≥ .162].

#### 5.2.3 Experiment 3

In a non-verbal episodic memory task, we presented black-and-white line drawings of items during the training phase (mostly from Snodgrass & Vanderwart, 1980) and coloured photographs of the same objects for recognition. These images were as dissimilar as possible to prevent participants from relying on perceptual matching to identify the target. We again manipulated semantic relatedness (related, unrelated) and episodic encoding strength (pairs presented once or five times). Items on semantically-related trials were drawn from the same semantic category (e.g., apple-orange). Other aspects of the procedure followed the description for Experiment 1 (see Fig. 4A for design and Appendix Table 3 for list of stimuli).

### 5.3 Results

Descriptive statistics are provided in Supplementary Table 5.

#### 5.3.1 Effects of relatedness and episodic strength on verbal recognition accuracy

Fig. 4C shows the key results. Patients showed poorer performance than controls in verbal recognition overall [Experiment 1: t(21) = 5.45, *p* < .001; Experiment 2: t(11.6) = 8.0; *p* < .001]. In Experiment 1, ANOVA was used to examine the effects of group, semantic relatedness (related vs. unrelated probe-target pairs) and episodic strength (episodic encoding weak vs. strong). This revealed main effects of semantic relatedness [F(1,21) = 49.63, *p* < .001] and episodic strength [F(1,21) = 7.80, *p* = .011]. There was a significant interaction between group and semantic relatedness [F(1,21) = 16.62, *p* = .001; Fig. 4A]: patients derived a larger benefit from the availability of pre-existing semantic links at encoding [patients: t(9) = 5.93, *p* > .001; controls: t(12) = 2.94, *p* = .024, Bonferroni-corrected], perhaps because they were less able than controls to find a way to link unrelated pairs during encoding. There was also a near–significant interaction between relatedness, episodic strength and group [F(1,21) = 4.26, *p* = .052]. Neither patients nor controls showed an effect of episodic strength in the unrelated condition [although the contrast approached significance for controls: t(12) = 2.48, *p* = .060; patients: t < 1, Bonferroni corrected for two comparisons]. In the related condition, controls showed better accuracy on episodic strong vs. weak trials [t(12) = 3.64, *p* = .009], while the patients remained insensitive to this manipulation [t(9) = 2.05, *p* = .140, Bonferroni corrected for two comparisons]. Moreover, episodic strength had no effect across groups in Experiment 2, when all of the trials were unrelated [main effect and interaction, F ≤ 2.7].

#### 5.3.2 Effects of presentation modality on accuracy

Fig. 4E shows key results. In Experiment 3, which employed pictures, patients were again less accurate than controls [t(21) = 6.19; *p* < .001]. In contrast to Experiment 1, there was no main effect of relatedness on picture recognition [F(1,21) = 2.46, *p* = .132], and no relatedness by group interaction [F < 1]. There was a main effect of episodic strength [F(1,21) = 24.08, *p* < .001], which did not differ across the groups [F < 1]. An analysis of modality (pictures in Experiment 3 vs. words in Experiment 1) and group (patients and controls) found main effects of group [better performance for controls, F (1,21) = 46.04, *p* < .001] and modality [better performance for pictures, F(1,21) = 4.63, *p* = .043] but no interaction [F < 1], indicating a multimodal deficit of comparable severity for words and pictures.

#### 5.3.3 Semantic error analysis

Since SA patients have difficulty controlling semantic retrieval to suit the task demands (Noonan et al., 2010), they may find it difficult to ignore semantic connections that are irrelevant for episodic memory (e.g., the distractor teacher for the encoded pair “school-cake”). We examined whether the patients were more likely than controls to choose semantically-related responses using ANOVA to compare related and unrelated trials, separately for each experiment and error type (expressed as a percentages of incorrect trials per condition). In Experiment 1 employing words, SEM errors (i.e., related in meaning but not previously presented) were the only error type selected more often by the patients [F(1,21) = 14.79, *p* = .001, Fig. 4D]. This pattern was not observed in Experiment 3 employing pictures [for SEM errors, there were no main effects of group and no interaction, F ≤ 2.41, *p* > .135]. It might be easier to reject novel distractor pictures – even those which are semantically-related – given the richness and distinctiveness of these stimuli.

#### 5.3.4 Proactive interference and perseveration errors

Proactive interference errors were coded when the correct response from a previous list was repeated (e.g., 1st list: party-children → “party-children”; 2nd list: party-basket → “party-children”), while perseveration errors were scored when the same incorrect response occurred across two lists (e.g., 1st list: party-children → “party-balloon”; 2nd list: party-basket → “party-balloon”). These errors were expressed as a percentage of incorrect trials in which the error was possible. In Experiment 1, patients made more proactive interference errors [t(21) = 4.02, *p* = .001] and perseverations [t(12.6) = 2.90, *p* = .011] than controls. All perseverations were semantically related to the probe. Similarly, in Experiment 2 employing unrelated words, patients made more proactive interference errors than controls [t(21) = 5.08; *p* < .001] but there were few perseverations in both groups and no group difference [t ≤ 1], consistent with the semantic origin of these errors in Experiment 1. In Experiment 3, when items were presented as pictures, there was no difference across groups in the rate of proactive interference [t(12.64) = 1.64, *p* = .125] and perseveration errors [t(9) = 2.17, *p* = .058].

#### 5.3.5 Confidence ratings

We used Linear Mixed Effects Models to examine the effects of trial-by-trial accuracy as well as experimental factors on confidence ratings, and to overcome missing data (i.e., controls without incorrect trials or patients without correct trials in particular conditions). Main effects and interaction terms were retained only if they improved the model fit. Allowing random intercepts per participant improved model fit in all analyses [χ^2^ (1) ≥ 3.84, *p* ≤ .05]. Key results are displayed in Fig. 4F with additional details in the Supplementary Materials. Interactions with group were interpreted by conducting separate multilevel models for patients and controls.

In the final model for Experiment 1 [-2LL = 4009.91], confidence ratings were predicted by response accuracy [F(1, 1451) = 88.07, *p* < .001]; relatedness of response [F(1, 1451) = 34.65, *p* < .001], episodic strength [F(1, 1449) = 23.30, *p* < .001], group [F(1, 24) = 7.76, *p* = .010] and the interaction between group and relatedness [F (1, 1451) = 4.6, *p* = .032]. Patients had disproportionately higher confidence in their episodic memory when they selected a semantically-related item [b = .27, F(1, 631) = 24.98, *p* < .001; Fig. 4F] relative to the controls [b = .13, F(1, 791) = 9.09, *p* = .003]. In Experiment 2, all probe-target pairs were semantically-unrelated; therefore, this experiment was not suited to investigating confidence for semantically-driven false memories. In Experiment 3 (episodic picture task), confidence did not show an interaction between group and relatedness of the response (there was a four-way interaction), while confidence in Experiment 4 (described below) did not show any interactions with group (see Supplementary Materials sections 1.1. and 1.2.). Analyses of the patient group confirmed that confidence was predicted by accuracy in all four experiments [Experiment 1: F(1,630) = 40.17, *p* < .001; Experiment 2: F(1,631) = 55.26, *p* < .001; Experiment 3: F(1,631) = 50.49, *p* < .001; Experiment 4: F(1,1150) = 44.9, *p* < .001], indicating that these participants were able to produce meaningful confidence ratings.

#### 5.3.6 Summary

Semantic links between probes and target at encoding supported episodic memory for the patients (Experiment 1 and 2), whereas the presence of semantic distractors and previously encoded memories (i.e., proactive interference) at retrieval elicited a disproportionate number of false episodic memories and perseverations (Experiment 1 and 2). Episodic deficits also arose when non-verbal material was used (Experiment 3) and patients were disproportionately confident when their response was congruent with existing semantic knowledge (Experiment 1).

## 6 Effects of episodic distractors on semantic decisions

### 6.1 Rationale

In the episodic memory tasks above, the patients relied more than controls on semantic links between probes and targets and they were vulnerable to false memories that reflected difficulties resolving competition between episodic and semantic representations. Next we established whether the patients’ difficulties reflected a failure to control semantic retrieval specifically, or if there were parallel deficits in supressing irrelevant episodic links when making semantic judgements. Unrelated items were paired to create episodic associations, and participants subsequently made semantic judgements to these items. On some trials, the probe and target had been previously presented as a pair, while on others, the probe was episodically-linked to a distractor. One participant (P8) was unable to take part in Experiment 4.

### 6.2 Method

Experiment 4 included two phases: *episodic training* and *semantic judgments*. During *episodic training,* participants pressed the arrow keys to indicate the location of an item on the screen, relative to another in the centre. In each session, there were four pairs of semantically-unrelated pictures presented consecutively; verbal labels were displayed underneath each picture and read aloud by the examiner. To check that the pairs had been encoded, participants were asked to recognize the episodic target alongside an unrelated foil (2AFC: e.g., “Was tea presented with money or dress?”). They were tested on three separate trials, employing different foils, both immediately and after a filled delay of twenty minutes. All participants were correct on both immediate and delayed recognition in at least two out of three trials.

**Fig. 5:**
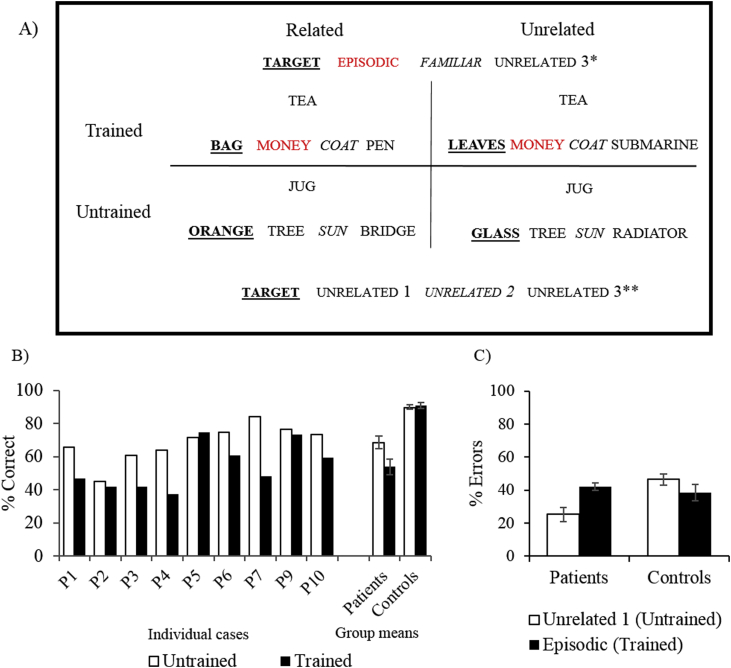
Experiment 4 (semantic judgement task with and without episodic distractors): design and key results. A) Experiment 4 design. Trained trials: probe associated with episodic distractor during training phase; Untrained trials: probe not presented during episodic training; Related trials: episodic distractor semantically related to target; Unrelated trials: episodic distractor unrelated to target. Response options: Target – semantically associated with probe; Episodic (trained trials only) – associated with the probe during episodic training; Familiar (trained trials only): associated with a different probe during episodic training; Unrelated: novel unrelated distractors (all distractors were unrelated in untrained trials). B) Effect of episodic training on semantic judgement. C) Percentage of errors that were episodically-associated with the probe, relative to selection of matched distractors on untrained trials. Error bars show SE of mean.

The *semantic judgment* task (Fig. 5A) immediately followed delayed recognition. There were eight probe words, including the four probes trained in the episodic training phase, plus four new and untrained ones. Each probe was presented on four different trials, with different semantically-related targets, producing a total of 32 trials. In half of the trials, the target was presented alongside a distractor that had been episodically-associated with the probe. In the other trials, none of the distractors had been presented in the episodic training phase. Additionally, in half the trials, this critical distractor was semantically-related to the target [LSA: M(SD) = .34 (.2); e.g., money with bag] but not the probe [LSA: M(SD) = .1 (.1) money with tea]. Consequently, the target might accrue activation from both the semantic link with the probe and the primed distractor. In the other trials, there was no semantic association between the target and the distractor [LSA: M(SD) = .08 (.09); e.g., money with leaves).

The target was presented alongside three distractors. On trials with episodic training, these were the episodic distractor, a familiar distractor that was associated with a different probe during episodic training and a novel unrelated distractor. On trials without episodic training, all distractors were unrelated [LSA: M(SD) = .08 (.08)]. The stimuli are provided in Appendix Table 4. The response options were presented visually and read aloud to the patients, who indicated their choice by pointing. This entire procedure was repeated on four different lists on separate sessions, providing 128 trials for analysis. Untrained trials on one list became trained trials in another, ensuring that differences between conditions could only be explained in terms of the effects of training. The order of trials and lists were randomized across participants. Prior to the semantic judgment task, participants were warned of the different task requirements and explicitly instructed and reminded over the course of the task to select words “related in meaning”. To ensure understanding of task instructions, the actual task was preceded in all sessions by two semantic judgment practice trials and explicit feedback were provided (a green tick as opposed to a red cross, when correct vs. incorrect). Participants were always correct in the practice trials and showed insight about their accuracy (see Supplementary Materials section 1.2. for confidence analysis) suggesting they understood the task instructions.

### 6.3 Results

Descriptive statistics are provided in Supplementary Table 5.

#### 6.3.1 Effect of episodic training on semantic judgments

Fig. 5B shows the key results. ANOVA examining the effects of episodic training, target-distractor relatedness and group revealed a main effect of episodic training [F(1,17) = 9.89, *p* = .006] and an episodic training by group interaction [F(1,17) = 13.32, *p* = .002]. There were fewer correct responses for episodically-trained trials in patients but not controls [patients: t(8) = −3.56, *p* = .014: controls: t < 1; Bonferroni corrected, Fig. 5B]. There was also a main effect of relatedness [F(1,17) = 29.24, *p* < .001] showing that both groups were more accurate when the target was semantically related to a distractor.

#### 6.3.2 Episodic error analysis

We compared selection of the episodic distractor on trials with episodic training with the matched unrelated distractor on trials without episodic training, with errors expressed as a percentage of incorrect trials. Key results are reported in Fig. 5C. There was a main effect of group [F(1,17) = 7.33, *p* = .015 and a significant interaction of error type by group [F(1,17) = 7.55, *p* = .014]: patients were more likely to choose the episodic distractor following training [patients: t(8) = 3.86, *p* = .01; controls: t(9) = −1.04, *p* = .6, Bonferroni corrected, see Fig. 5C].

## 7 Correlation between semantic and episodic performance

**Fig. 6:**
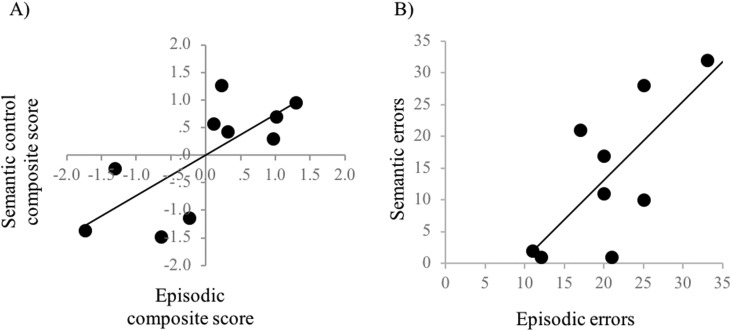
Correlations between semantic and episodic performance.

The semantic control composite score (see above) and an episodic composite score derived from overall accuracy in Experiments 1, 2 and 3 were highly correlated [r = .736, *p* = .015, Fig. 6A]. Similarly, there was a strong correlation between the number of semantic and episodic errors [from Experiment 1 and 4 respectively, r = .729, *p* = .026, Fig. 6B]. This suggests that semantic control difficulties are highly associated with episodic memory performance, as is the capacity to avoid errors driven by both irrelevant episodic and semantic information.

## 8 Discussion

This study investigated deficits of episodic memory in patients with multimodal semantic impairment following stroke aphasia (cf. SA). These individuals have deficient executive control over semantic information, as opposed to a loss of conceptual knowledge, following lesions in frontal and/or temporoparietal regions (Jefferies and Lambon Ralph, 2006, Noonan et al., 2010, Thompson et al., 2015). In the current sample, the lesion overlay was focussed on LIFG, a key region for semantic control, and all patients had damage to this region. While past studies of these patients have focussed exclusively on deficits in semantic tasks, we might expect parallel deficits in episodic memory since functional neuroimaging studies have implicated LIFG in controlled retrieval across both semantic and episodic tasks. In line with this hypothesis, we found patients had difficulty retrieving information in a flexible fashion appropriate to the circumstances in both episodic and semantic tasks. In the semantic domain, the patients struggled to understand non-dominant interpretations of ambiguous words as well as non-canonical uses of objects presented as pictures (cf. Corbett et al., 2011, Noonan et al., 2010). In the episodic domain, the patients were impaired at paired-associate learning tasks, particularly when the target was presented alongside a recent item from another trial or a distractor that was strongly-related to the probe, causing interference. Like the semantic deficit, this impairment of episodic memory was multimodal, affecting paired-associate tasks presented using words or pictures, supporting the view that shared control processes interact with heteromodal episodic and semantic representations in the hippocampus and anterior temporal lobes.

The patients relied on well-established semantic links during episodic encoding. They had difficulty forming associations ‘on the fly’ between words that were not already related – and consequently, their semantic control deficit increased rather than reduced their reliance on semantic information in episodic tasks. Their episodic retrieval was inappropriately driven by semantic connections, leading to the intrusion of irrelevant information (i.e., false recognition of semantically-related distracters). This resembles the pattern for semantic judgements; patients also had difficulty correctly identifying synonyms when the target word was presented alongside a strong associate that acted as a distracter (e.g., piece with slice and cake). Patients' confidence in their episodic memory was strongly driven by the semantic relationship between the response and probe, suggesting they had difficulty appropriately focussing on the strength of task-relevant as opposed to irrelevant information to evaluate their memory. This impairment is likely to have a significant impact on everyday functioning, since patients have difficulty separating strong semantic signals from representations of past events.

The patients also showed increased proactive interference, suggesting they had weak inhibition over competing episodic memories. This pattern would be expected if the same neurocognitive mechanisms support episodic and semantic selection. To confirm this interpretation, we demonstrated that presenting pairs of unrelated words to create episodic associations generated interference during subsequent semantic judgements involving the same items. The patients’ difficulties did not simply reflect the impaired application of semantic knowledge to promote successful episodic encoding and retrieval. Instead, they had difficulty regulating activation in *both* memory systems and generating appropriate cognitive states when these two sets of memory representations were in conflict. The patients also showed similar effects of cueing on episodic and semantic retrieval. Episodic memory was improved by the provision of progressive phonological cues indicating that the patients were able to encode and retain information in episodic memory, yet they had difficulty focussing retrieval on *relevant* information when the task was relatively unconstrained. Similar effects of semantic cueing have been observed in picture naming (Jefferies et al., 2008, Soni et al., 2009) and comprehension tasks (Noonan et al., 2010), including in the current patients. In sum, our findings suggest that shared mechanisms are responsible for focussing cognition on currently-relevant memory representations, especially in the face of competition from strongly-encoded yet irrelevant information, in both episodic and semantic tasks. This necessity to constrain retrieval is reduced when the task provides strong cues to retrieval that reduce competition and the need to internally shape retrieval.

Our findings have important implications for neuroscientific accounts of memory retrieval. Most neuroimaging and neuropsychological studies to date have examined manipulations of either episodic *or* semantic tasks, and have not directly compared effects of control demands across these domains. This study therefore provides new insights into how these representations interact in ways that both support and impair performance. Distinct heteromodal LTM representations supporting generalised and unique aspects of experience are thought to lie in adjacent regions of ventral ATL and hippocampus (McClelland et al., 1995, OReilly et al., 2014), and these sources of semantic and episodic information are likely to be highly interactive. Learning benefits from existing knowledge that is coherent with new experiences (Bartlett, 1932, Craik and Tulving, 1975, Van Kesteren et al., 2012). Also, intact semantic knowledge can support episodic memory in amnesic patients with selective hippocampal lesions (Verfaellie, Koseff, & Alexander, 2000) and new episodic learning is influenced by degraded semantic knowledge in semantic dementia (Mayberry, Sage, Ehsan, & Lambon Ralph, 2011). The activation of conceptual representations at the point of retrieval can then give rise to competition between these systems. The patients relied to a greater extent than the healthy controls on semantic representations to aid episodic learning, presumably because control processes are critical to establish new links that are unsupported by past experience. By the same token, the patients were vulnerable to false memories driven by irrelevant semantic associations, presumably because control processes also play a critical role in selecting memory representations to suit the current demands of the task. Irrespective of the type of memory, the patients were overly influenced by the most dominant, activated form of information (episodic or semantic).

In addition, while neuroimaging studies of healthy volunteers have demonstrated a role for LIFG in executive aspects of both semantic and episodic tasks (in separate studies), the current work adds weight to the view that LIFG plays a *critical* role in memory control across domains, since neuropsychological studies are causal and not correlational. The neuroimaging findings of Badre and colleagues have linked distinct regions of LIFG to (i) controlled retrieval and (ii) post-retrieval selection, across semantic and episodic memory tasks (Badre and Wagner, 2007, Barredo et al., 2015). Mid-to-posterior LIFG, damaged in every patient in our sample, is thought to contribute to the resolution of competition between activated representations in both episodic and semantic judgements (Badre and Wagner, 2005, Badre and Wagner, 2007, Barredo et al., 2015) and this region also makes a crucial contribution to lexical selection and phonological tasks (Gold and Buckner, 2002, Hirshorn and Thompson-Schill, 2006, Poldrack et al., 1999). LIFG is known to be engaged in situations in which recently-activated information is irrelevant to the current task, such as in the recent negatives paradigm (Badre and Wagner, 2005, Jonides et al., 1998). The effect of distracters and cues in episodic and semantic memory tasks, and the frequency of perseverations and interference errors, can be explained in terms of a deficit in selecting relevant semantic and episodic representations. Mid-to-anterior parts of LIFG are proposed to have a more specific role in memory retrieval, assisting with the recovery of weakly-encoded semantic and episodic information (Barredo et al., 2015). There is less clear-cut evidence of this deficit: although we manipulated episodic encoding strength, the patients showed a smaller effect of this variable than the controls, at least when semantic relationships were also available at encoding. However, the patients’ large lesions do not allow us to separately examine the contributions of anterior and posterior aspects of LIFG.

Most neuroimaging and neuropsychological studies of memory control have employed verbal stimuli (but see Turriziani et al., 2010, Krieger-Redwood et al., 2015): the current work is therefore also important in demonstrating that shared neurocognitive processes support memory control for *non-verbal* episodic and semantic tasks (Corbett et al., 2011, Krieger-Redwood and Jefferies, 2014, Thompson et al., 2015). These results are explicable within a framework in which modality-general control processes (drawing on LIFG and other temporo-parietal regions) interact with heteromodal representations captured within ATL (a key hub for semantic representations) and hippocampus (the episodic ‘store’). However, differences between the verbal and non-verbal tasks (e.g., in the effect of semantic encoding and distraction) also place constraints on this theoretical framework. While the verbal episodic memory task showed a strong positive effect of semantic relatedness at encoding, and significant disruption from semantically-related distracters, the picture-based task showed neither of these effects. One possibility is that semantic-episodic interactions are stronger for verbal tasks, in line with the proposal that pictures gain privileged access to the hippocampus via the ventral visual stream (Baddeley and Hitch, 2017, Graham et al., 2010). As a consequence, both the positive and negative consequences of semantic involvement in paired associate learning may be greater for verbal stimuli.

### 8.1 Limitations and future directions

Our past work has pointed to roles for both posterior middle temporal gyrus (pMTG) and dorsal angular gyrus (dAG) in semantic control (Noonan et al., 2013). The contribution of these regions to controlled episodic retrieval is yet to be established, but would be predicted given the large-scale distributed network that LIFG participates in. Both pMTG and dAG are commonly damaged in patients with aphasia following left hemisphere strokes, although unlike LIFG, these regions were not universally affected in the current sample. Although our data support the hypothesis of a critical role of LIFG in memory control (Badre and Wagner, 2007, Barredo et al., 2015), the current study cannot provide incontrovertible evidence that LIFG – and no other sites – within MCA-territory infarcts support controlled retrieval from episodic and semantic memory. Future studies could address this issue by comparing episodic performance after LIFG and other lesions (either in clinical groups or through the use of inhibitory TMS). In the current study we have shown that patients with LIFG lesions have difficulty controlling competition within and between episodic and semantic memory. Our focus is on shared components at the cognitive level, and the extent to which this pattern extends to patients with left hemisphere stroke outside IFG remains debatable.

We have previously shown a double dissociation in semantic cognition between patients with SA and people with semantic dementia (Jefferies & Lambon Ralph, 2006). SA patients show impaired control over semantic retrieval, while semantic dementia is linked to degraded conceptual knowledge. It would be useful to confirm there is a similar double dissociation in episodic memory between SA and patients with hippocampal lesions, who might be expected to have impaired episodic memory yet intact memory control processes. Future studies could also test if stroke survivors who have a cognitive profile not compatible with SA – such as those with relatively specific phonological deficits – show intact retrieval of episodic memories.

### 8.2 Conclusions

We observed similar control deficits in episodic and semantic tasks in our patient sample with LIFG lesions. These results support the hypothesis that common control processes across episodic and semantic memory focus retrieval on currently-relevant representations, especially in the face of competition from strongly-encoded yet irrelevant information. There were parallel effects of strong competitors and cueing, plus a multi-modal deficit in both semantic and episodic memory. The patients experienced false episodic memories driven by the inappropriate retrieval of semantic associations and, similarly, recent experience inappropriately influenced the patients’ semantic judgements. This indicates that episodic representations of recent events and semantic representations of common elements of experience are both utilised to support episodic and semantic judgements. Control processes normally play a crucial role in allowing us to weight these sources of information to suit the circumstances.

## Funding

SS was supported by a grant from the Stroke Association [TSA/12/02]. EJ was supported by a grant from the European Research Council [SEMBIND – 283530]. The work was also part-funded by the Wellcome Trust [ref: 105624] through the Centre for Chronic Diseases and Disorders (C2D2) at the University of York. MALR was supported by an Medical Research Council programme grant [MR/J004146/1].

## Appendix A Supplementary data

Appendix Multimedia component 1

Raw data Multimedia component 2
