# Supplementary material for: Shared processes resolve competition within and between episodic and semantic memory: Evidence from patients with LIFG lesions
Source: Cortex. 2018 Nov;108:127–43. doi: 10.1016/j.cortex.2018.07.007 (PMC6238079; doi:10.1016/j.cortex.2018.07.007)
Supplement: Appendix [file mmc1.docx]

Appendix

Table 1: List of stimuli for Experiment 1

| List |  |  |  | Distractors | | | Relatedness | Episodic strength |
| --- | --- | --- | --- | --- | --- | --- | --- | --- |
|  |  | Probe | Target | SEM+EP | SEM | EP |  |  |
| A |  | party | **children** | *cake* | balloon | basket | related | strong |
|  |  | fruit | **cake** | *basket* | loaf | bus | related | strong |
|  |  | school | **bus** | *children* | teacher | cake | related | strong |
|  |  | bicycle | **basket** | *bus* | car | children | related | strong |
|  |  | violin | **orchestra** | *conductor* | trumpet | bus | related | weak |
|  |  | baton | **conductor** | *orchestra* | relay | basket | related | weak |
|  |  | war | **army** | *navy* | soldier | cake | related | weak |
|  |  | officers | **navy** | *army* | police | children | related | weak |
| B |  | party | **basket** | *children* | balloon | bus | unrelated | strong |
|  |  | fruit | **bus** | *cake* | loaf | children | unrelated | strong |
|  |  | school | **cake** | *bus* | teacher | basket | unrelated | strong |
|  |  | bicycle | **children** | *basket* | car | cake | unrelated | strong |
|  |  | violin | **army** | *orchestra* | trumpet | bus | unrelated | weak |
|  |  | baton | **navy** | *conductor* | relay | children | unrelated | weak |
|  |  | war | **conductor** | *army* | soldier | basket | unrelated | weak |
|  |  | officers | **orchestra** | *navy* | police | cake | unrelated | weak |
| C |  | christmas | **bottle** | *turkey* | present | cow | unrelated | weak |
|  |  | chicken | **glass** | *wing* | hen | donkey | unrelated | weak |
|  |  | fairy | **donkey** | *angel* | wand | sea | unrelated | strong |
|  |  | beach | **cow** | *sea* | sand | angel | unrelated | strong |
|  |  | water | **turkey** | *glass* | tap | cow | unrelated | weak |
|  |  | wine | **wing** | *bottle* | beer | donkey | unrelated | weak |
|  |  | milk | **angel** | *cow* | cream | sea | unrelated | strong |
|  |  | farm | **sea** | *donkey* | yard | angel | unrelated | strong |
| D |  | christmas | **turkey** | *angel* | present | sea | related | weak |
|  |  | chicken | **wing** | *turkey* | hen | angel | related | weak |
|  |  | fairy | **angel** | *wing* | wand | donkey | related | strong |
|  |  | beach | **sea** | *donkey* | sand | cow | related | strong |
|  |  | water | **glass** | *sea* | tap | donkey | related | weak |
|  |  | wine | **bottle** | *glass* | beer | cow | related | weak |
|  |  | milk | **cow** | *bottle* | cream | angel | related | strong |
|  |  | farm | **donkey** | *cow* | yard | sea | related | strong |
| E |  | lunch | **soup** | *juice* | sandwich | rain | related | strong |
|  |  | vegetable | **juice** | *soup* | broth | rain | related | strong |
|  |  | stone | **picnic** | *cliff* | rock | juice | unrelated | strong |
|  |  | bullet | **rain** | *gun* | pistol | picnic | unrelated | strong |
|  |  | apple | **seed** | *tree* | orange | soup | related | weak |
|  |  | pear | **tree** | *seed* | cider | soup | related | weak |
|  |  | puddle | **cliff** | *rain* | drizzle | picnic | unrelated | weak |
|  |  | bench | **gun** | *picnic* | chair | juice | unrelated | weak |
| F |  | lunch | **picnic** | *soup* | sandwich | tree | related | weak |
|  |  | vegetable | **soup** | *juice* | broth | cliff | related | weak |
|  |  | stone | **rain** | *cliff* | rock | gun | unrelated | weak |
|  |  | bullet | **juice** | *gun* | pistol | seed | unrelated | weak |
|  |  | apple | **tree** | *seed* | orange | cliff | related | strong |
|  |  | pear | **seed** | *tree* | cider | gun | related | strong |
|  |  | puddle | **gun** | *rain* | drizzle | tree | unrelated | strong |
|  |  | bench | **cliff** | *picnic* | chair | seed | unrelated | strong |
| G |  | wedding | **dress** | *guest* | bride | biscuit | related | strong |
|  |  | hotel | **guest** | *holiday* | bedroom | dress | related | strong |
|  |  | church | **holiday** | *steeple* | vicar | dress | unrelated | strong |
|  |  | camera | **biscuit** | *photo* | picture | guest | unrelated | strong |
|  |  | garden | **grass** | *swing* | pond | guest | related | weak |
|  |  | playground | **swing** | *grass* | slide | holiday | related | weak |
|  |  | sun | **photo** | *dress* | cloud | biscuit | unrelated | weak |
|  |  | coffee | **steeple** | *biscuit* | cappuccino | holiday | unrelated | weak |
| H |  | wedding | **guest** | *dress* | bride | grass | related | weak |
|  |  | hotel | **holiday** | *guest* | bedroom | steeple | related | weak |
|  |  | church | **biscuit** | *steeple* | vicar | swing | unrelated | weak |
|  |  | camera | **dress** | *photo* | picture | grass | unrelated | weak |
|  |  | garden | **swing** | *grass* | pond | steeple | related | strong |
|  |  | playground | **grass** | *swing* | slide | photo | related | strong |
|  |  | sun | **steeple** | *holiday* | cloud | photo | unrelated | strong |
|  |  | coffee | **photo** | *biscuit* | cappuccino | swing | unrelated | strong |

Legend. Related and Unrelated = probe paired with a semantically related/unrelated target at encoding; Strong = repeated 5 times at encoding; Weak = presented only once at encoding; SEM: novel and semantically related to the probe; SEM+EP: semantically related to the probe and target word for another probe; EP: target on a different trial but not semantically related to the probe.

Table 2: List of stimuli Experiment 2

| List |  |  | Distractors | | | Episodic strength |
| --- | --- | --- | --- | --- | --- | --- |
|  | Probe | Target | EP 1 | Novel | EP 2 |  |
| A | party | **mineral** | storm | cat | sponge | strong |
|  | fruit | **storm** | sponge | tap | duck | strong |
|  | school | **duck** | mineral | sand | storm | strong |
|  | bicycle | **sponge** | duck | present | mineral | strong |
|  | violin | **rose** | shirt | beer | duck | weak |
|  | baton | **shirt** | rose | tiger | sponge | weak |
|  | war | **fur** | bottle | cream | storm | weak |
|  | officers | **bottle** | fur | hammer | mineral | weak |
| B | party | **sponge** | mineral | cat | duck | strong |
|  | fruit | **duck** | storm | tap | mineral | strong |
|  | school | **storm** | duck | sand | sponge | strong |
|  | bicycle | **mineral** | sponge | present | storm | strong |
|  | violin | **fur** | rose | beer | duck | weak |
|  | baton | **bottle** | shirt | tiger | mineral | weak |
|  | war | **shirt** | fur | cream | sponge | weak |
|  | officers | **rose** | bottle | hammer | storm | weak |
| C | christmas | **rabbit** | volcano | car | pencil | weak |
|  | chicken | **poster** | mail | teacher | number | weak |
|  | fairy | **number** | wheel | trumpet | college | strong |
|  | beach | **pencil** | college | graph | wheel | strong |
|  | water | **volcano** | poster | police | pencil | weak |
|  | wine | **mail** | rabbit | balloon | number | weak |
|  | milk | **wheel** | pencil | relay | college | strong |
|  | farm | **college** | number | kiss | wheel | strong |
| D | christmas | **volcano** | wheel | car | college | weak |
|  | chicken | **mail** | volcano | teacher | wheel | weak |
|  | fairy | **wheel** | mail | trumpet | number | strong |
|  | beach | **college** | number | graph | pencil | strong |
|  | water | **poster** | college | police | number | weak |
|  | wine | **rabbit** | poster | balloon | pencil | weak |
|  | milk | **pencil** | rabbit | relay | wheel | strong |
|  | farm | **number** | pencil | kiss | college | strong |
| E | lunch | **court** | star | slide | queen | strong |
|  | vegetable | **star** | court | cloud | queen | strong |
|  | stone | **birth** | minister | cappuccino | star | strong |
|  | bullet | **queen** | kite | pond | birth | strong |
|  | apple | **poet** | rectangle | vicar | court | weak |
|  | pear | **rectangle** | poet | bride | court | weak |
|  | puddle | **minister** | queen | picture | birth | weak |
|  | bench | **kite** | birth | rock | star | weak |
| F | lunch | **birth** | court | slide | rectangle | weak |
|  | vegetable | **court** | star | cloud | minister | weak |
|  | stone | **queen** | minister | cappuccino | kite | weak |
|  | bullet | **star** | kite | pond | poet | weak |
|  | apple | **rectangle** | poet | vicar | minister | strong |
|  | pear | **poet** | rectangle | bride | kite | strong |
|  | puddle | **kite** | queen | picture | rectangle | strong |
|  | bench | **minister** | birth | rock | poet | strong |
| G | wedding | **turtle** | rubber | lion | river | strong |
|  | hotel | **rubber** | skull | pliers | turtle | strong |
|  | church | **skull** | ear | orange | turtle | strong |
|  | camera | **river** | horse | cider | rubber | strong |
|  | garden | **island** | screw | hair | rubber | weak |
|  | playground | **screw** | island | pistol | skull | weak |
|  | sun | **horse** | turtle | chain | river | weak |
|  | coffee | **ear** | river | nurse | skull | weak |
| H | wedding | **rubber** | turtle | lion | island | weak |
|  | hotel | **skull** | rubber | pliers | ear | weak |
|  | church | **river** | ear | orange | screw | weak |
|  | camera | **turtle** | horse | cider | island | weak |
|  | garden | **screw** | island | hair | ear | strong |
|  | playground | **island** | screw | pistol | horse | strong |
|  | sun | **ear** | skull | chain | horse | strong |
|  | coffee | **horse** | river | nurse | screw | strong |

Legend: Strong = repeated 5 times at encoding; Weak = presented only once at encoding; EP 1 and 2 = target on a different trial but not semantically related to the probe.

Table 3: List of stimuli Experiment 3

| List |  |  | Distractors | | | | Relatedness | Episodic strength |
| --- | --- | --- | --- | --- | --- | --- | --- | --- |
|  | Probe | Target | SEM+EP | SEM | | EP |  |  |
| A | pen | **notebook** | *scissors* | pencil | | button | related | strong |
|  | needle | **scissors** | *button* | spool | | school uniform | related | strong |
|  | teacher | **school uniform** | *notebook* | textbooks | | scissors | related | strong |
|  | shirt | **button** | *school uniform* | trousers | | notebook | related | strong |
|  | violin | **piano** | *conductor* | trumpet | | school uniform | related | weak |
|  | saxophone | **conductor** | *piano* | musical score | | button | related | weak |
|  | bomb | **war helmet** | *machine gun* | soldier | | scissors | related | weak |
|  | panzer | **machine gun** | *war helmet* | cannon | | notebook | related | weak |
| B | pen | **button** | *notebook* | pencil | | school uniform | unrelated | strong |
|  | needle | **school uniform** | *scissors* | spool | | notebook | unrelated | strong |
|  | teacher | **scissors** | *school uniform* | | textbooks | button | unrelated | strong |
|  | shirt | **notebook** | *button* | trousers | | scissors | unrelated | strong |
|  | violin | **war helmet** | *piano* | trumpet | | school uniform | unrelated | weak |
|  | saxophone | **machine gun** | *conductor* | musical score | | notebook | unrelated | weak |
|  | bomb | **conductor** | *war helmet* | soldier | | button | unrelated | weak |
|  | panzer | **piano** | *machine gun* | cannon | | scissors | unrelated | weak |
| C | plant | **glass** | *watering can* | flower pot | | cow | unrelated | weak |
|  | lawn mower | **wine glass** | *grass* | shears | | well | unrelated | weak |
|  | tree | **well** | *roots* | leaf | | bottle | unrelated | strong |
|  | water | **cow** | *bottle* | tap | | roots | unrelated | strong |
|  | wine | **watering can** | *wine glass* | corkscrew | | cow | unrelated | weak |
|  | bowl | **grass** | *glass* | cup | | well | unrelated | weak |
|  | milk | **roots** | *cow* | yoghurt | | bottle | unrelated | strong |
|  | barn | **bottle** | *well* | tractor | | roots | unrelated | strong |
| D | plant | **watering can** | *roots* | flower pot | | bottle | related | weak |
|  | lawn mower | **grass** | *watering can* | shears | | roots | related | weak |
|  | tree | **roots** | *grass* | leaf | | well | related | strong |
|  | water | **bottle** | *well* | tap | | cow | related | strong |
|  | wine | **wine glass** | *bottle* | corkscrew | | well | related | weak |
|  | bowl | **glass** | *wine glass* | cup | | cow | related | weak |
|  | milk | **cow** | *glass* | yoghurt | | roots | related | strong |
|  | barn | **well** | *cow* | tractor | | bottle | related | strong |
| E | arm | **hand** | *eye* | foot | | drum | related | strong |
|  | ear | **eye** | *hand* | nose | | drum | related | strong |
|  | chick | **finger** | *chicken* | eggs | | eye | unrelated | strong |
|  | bullet | **drum** | *gun* | shotgun | | finger | unrelated | strong |
|  | apple | **banana** | *cherry* | grapes | | hand | related | weak |
|  | strawberry | **cherry** | *banana* | pineapple | | hand | related | weak |
|  | harp | **chicken** | *drum* | accordion | | finger | unrelated | weak |
|  | ring | **gun** | *finger* | necklace | | eye | unrelated | weak |
| F | arm | **finger** | *hand* | foot | | cherry | related | weak |
|  | ear | **hand** | *eye* | nose | | chicken | related | weak |
|  | chick | **drum** | *chicken* | eggs | | gun | unrelated | weak |
|  | bullet | **eye** | *gun* | shotgun | | banana | unrelated | weak |
|  | apple | **cherry** | *banana* | grapes | | chicken | related | strong |
|  | strawberry | **banana** | *cherry* | pineapple | | gun | related | strong |
|  | harp | **gun** | *drum* | accordion | | cherry | unrelated | strong |
|  | ring | **chicken** | *finger* | necklace | | banana | unrelated | strong |
| G | rocking chair | **chair** | *lamp* | sofa | | dress | related | strong |
|  | light bulb | **lamp** | *ceiling lamp* | light switch | | chair | related | strong |
|  | zebra | **ceiling lamp** | *lion* | monkey | | chair | unrelated | strong |
|  | car | **dress** | *truck* | motorbike | | lamp | unrelated | strong |
|  | corn | **asparagus** | *artichoke* | celery | | lamp | related | weak |
|  | pumpkin | **artichoke** | *asparagus* | carrot | | ceiling lamp | related | weak |
|  | table | **truck** | *chair* | desk | | dress | unrelated | weak |
|  | waistcoat | **lion** | *dress* | coat | | ceiling lamp | unrelated | weak |
| H | rocking chair | **lamp** | *chair* | sofa | | asparagus | related | weak |
|  | light bulb | **ceiling lamp** | *lamp* | light switch | | lion | related | weak |
|  | zebra | **dress** | *lion* | monkey | | artichoke | unrelated | weak |
|  | car | **chair** | *truck* | motorbike | | asparagus | unrelated | weak |
|  | corn | **artichoke** | *asparagus* | celery | | lion | related | strong |
|  | pumpkin | **asparagus** | *artichoke* | carrot | | truck | related | strong |
|  | table | **lion** | *ceiling lamp* | desk | | truck | unrelated | strong |
|  | waistcoat | **truck** | *dress* | coat | | artichoke | unrelated | strong |

Legend. Related and Unrelated = probe paired with a semantically related/unrelated target at encoding; Strong = repeated 5 times at encoding; Weak = presented only once at encoding; SEM: novel and semantically related to the probe; SEM+EP: semantically related to the probe and target word for another probe; EP: target on a different trial but not semantically related to the probe.

Table 4: List of stimuli Experiment 4

| List | |  |  | Distractors | | | Relatedness | Episodic training | |
| --- | --- | --- | --- | --- | --- | --- | --- | --- | --- |
|  |  | Probe | Target | EPI/UNR1 | FAM | UNR2 |  | List 1 | List 2 |
| D | A | bicycle | **oil** | petrol | *pill* | duck | related | trained | untrained |
|  |  | bicycle | **pump** | petrol | *pill* | bacon | related | trained | untrained |
|  |  | bicycle | **spokes** | petrol | *pill* | plant | unrelated | trained | untrained |
|  |  | bicycle | **shed** | petrol | *pill* | mushroom | unrelated | trained | untrained |
|  |  | plate | **fish** | sea | *petrol* | cactus | related | trained | untrained |
|  |  | plate | **cod** | sea | *petrol* | torch | related | trained | untrained |
|  |  | plate | **lid** | sea | *petrol* | badge | unrelated | trained | untrained |
|  |  | plate | **tin** | sea | *petrol* | porch | unrelated | trained | untrained |
|  |  | soap | **tablet** | pill | *yellow* | tree | related | trained | untrained |
|  |  | soap | **water** | pill | *yellow* | missile | related | trained | untrained |
|  |  | soap | **brush** | pill | *yellow* | cliff | unrelated | trained | untrained |
|  |  | soap | **face** | pill | *yellow* | moose | unrelated | trained | untrained |
|  |  | lipstick | **purple** | yellow | *sea* | fennel | related | trained | untrained |
|  |  | lipstick | **pink** | yellow | *sea* | cork | related | trained | untrained |
|  |  | lipstick | **collar** | yellow | *sea* | sofa | unrelated | trained | untrained |
|  |  | lipstick | **powder** | yellow | *sea* | duvet | unrelated | trained | untrained |
| B |  | jug | **orange** | tree | *sun* | bridge | related | untrained | trained |
|  |  | jug | **flowers** | tree | *sun* | oyster | related | untrained | trained |
|  |  | jug | **glass** | tree | *sun* | radiator | unrelated | untrained | trained |
|  |  | jug | **pint** | tree | *sun* | parachute | unrelated | untrained | trained |
|  |  | scarf | **rain** | sun | *tree* | teeth | related | untrained | trained |
|  |  | scarf | **wind** | sun | *tree* | jeep | related | untrained | trained |
|  |  | scarf | **knot** | sun | *tree* | keyboard | unrelated | untrained | trained |
|  |  | scarf | **knit** | sun | *tree* | zoo | unrelated | untrained | trained |
|  |  | doll | **dress** | coat | *money* | boat | related | untrained | trained |
|  |  | doll | **rag** | coat | *money* | seal | related | untrained | trained |
|  |  | doll | **plastic** | coat | *money* | worm | unrelated | untrained | trained |
|  |  | doll | **sister** | coat | *money* | sunset | unrelated | untrained | trained |
|  |  | tea | **bag** | money | *coat* | pen | related | untrained | trained |
|  |  | tea | **shop** | money | *coat* | blouse | related | untrained | trained |
|  |  | tea | **leaves** | money | *coat* | submarine | unrelated | untrained | trained |
|  |  | tea | **spoon** | money | *coat* | train | unrelated | untrained | trained |
|  | C | cage | **prison** | court | *cake* | notebook | related | trained | untrained |
|  |  | cage | **imprisonment** | court | *cake* | foam | related | trained | untrained |
|  |  | cage | **monkey** | court | *cake* | cushion | unrelated | trained | untrained |
|  |  | cage | **box** | court | *cake* | pretzel | unrelated | trained | untrained |
|  |  | robe | **king** | castle | *medicine* | car | related | trained | untrained |
|  |  | robe | **queen** | castle | *medicine* | tobacco | related | trained | untrained |
|  |  | robe | **priest** | castle | *medicine* | fist | unrelated | trained | untrained |
|  |  | robe | **overcoat** | castle | *medicine* | cello | unrelated | trained | untrained |
|  |  | tweezers | **doctor** | medicine | *castle* | bin | related | trained | untrained |
|  |  | tweezers | **hospital** | medicine | *castle* | road | related | trained | untrained |
|  |  | tweezers | **skin** | medicine | *castle* | dwarf | unrelated | trained | untrained |
|  |  | tweezers | **lash** | medicine | *castle* | shell | unrelated | trained | untrained |
|  |  | penguin | **chocolate** | cake | *court* | gym | related | trained | untrained |
|  |  | penguin | **eggs** | cake | *court* | shop | related | trained | untrained |
|  |  | penguin | **suit** | cake | *court* | coin | unrelated | trained | untrained |
|  |  | penguin | **wing** | cake | *court* | pipe | unrelated | trained | untrained |
| D |  | candle | **chapel** | pope | *aeroplane* | stapler | related | untrained | trained |
|  |  | candle | **church** | pope | *aeroplane* | calf | related | untrained | trained |
|  |  | candle | **bottle** | pope | *aeroplane* | park | unrelated | untrained | trained |
|  |  | candle | **match** | pope | *aeroplane* | parcel | unrelated | untrained | trained |
|  |  | bag | **baggage** | aeroplane | *pope* | chimp | related | untrained | trained |
|  |  | bag | **luggage** | aeroplane | *pope* | worm | related | untrained | trained |
|  |  | bag | **leather** | aeroplane | *pope* | lawn | unrelated | untrained | trained |
|  |  | bag | **golf** | aeroplane | *pope* | river | unrelated | untrained | trained |
|  |  | sword | **pistol** | policeman | *parrot* | hay | related | untrained | trained |
|  |  | sword | **gun** | policeman | *parrot* | honey | related | untrained | trained |
|  |  | sword | **silver** | policeman | *parrot* | tooth | unrelated | untrained | trained |
|  |  | sword | **edge** | policeman | *parrot* | tinsel | unrelated | untrained | trained |
|  |  | pillow | **feather** | parrot | *policeman* | chalk | related | untrained | trained |
|  |  | pillow | **down** | parrot | *policeman* | nest | related | untrained | trained |
|  |  | pillow | **fight** | parrot | *policeman* | hippie | unrelated | untrained | trained |
|  |  | pillow | **sheets** | parrot | *policeman* | broom | unrelated | untrained | trained |

Legend: Untrained trials on one list became trained trials in another, ensuring that differences between conditions could only be explained in terms of the effects of training. Related and Unrelated = EPI/UNR1 distractors semantically related or unrelated with target; Trained = probe episodically-associated with episodic distractor during episodic training; Untrained = probe not presented during episodic training; EPI = episodic distractor associated with the probe during episodic training; FAM = associated with a different probe during episodic training; UNR1, 2, 3 = novel unrelated distractors
