# Supplementary material for: Shared processes resolve competition within and between episodic and semantic memory: Evidence from patients with LIFG lesions
Source: Cortex. 2018 Nov;108:127–43. doi: 10.1016/j.cortex.2018.07.007 (PMC6238079; doi:10.1016/j.cortex.2018.07.007)
Supplement: Supplementary materials [file mmc3.docx]

Supplementary materials

Supplementary Table 1. Demographics

| Patient ID | Age | Gender | Education* | Years since CVA | Type of Aphasia |
| --- | --- | --- | --- | --- | --- |
| P1 | 60 | F | 18 | 6 | Global |
| P2 | 77 | M | 15 | 6 | Mixed Transcortical |
| P3 | 59 | F | 16 | 8 | Global |
| P4 | 66 | M | 15 | 23 | Mixed Transcortical |
| P5 | 58 | F | 18 | 6 | Transcortical Sensory/Anomic |
| P6 | 57 | M | 18 | 13 | Transcortical Sensory |
| P7 | 65 | M | 16 | 6 | Broca |
| P8 | 70 | F | 16 | 10 | Transcortical Sensory/Anomic |
| P9 | 77 | F | 16 | 4 | Anomic |
| P10 | 39 | F | 16 | 7 | Transcortical Sensory/Anomic |
| Mean | 62.8 | 6/10 females | 16.4 | 8.9 |  |
| SD | 11.2 |  | 1.2 | 5.6 |  |
| * Age left education, CVA: Cerebrovascular accident | | | | | |

Supplementary Table 2. Patients lesions analysis

|  |  |  |  |  |  |  |  |  |  |  |  |  |  |  |  |  |  |  |
| --- | --- | --- | --- | --- | --- | --- | --- | --- | --- | --- | --- | --- | --- | --- | --- | --- | --- | --- |
| Patient ID | Lesion size** | Frontal | | | | | | | Parietal | | Temporal | | | | | | | |
|  |  | SMA/PMC | FP | DLPFC | | ant-IFG | mid-IFG | post-IFG | SMG | AnG | pMTG | TP | STG | MTG | ITG | FuG | PHG | Hpc |
|  |  | BA | BA | BA | BA | BA | BA | BA | BA | BA | BA | BA | BA | BA | BA | BA | BA | BA |
|  |  | 6 | 10 | 9 | 46 | 47 | 45 | 44 | 40 | 39 | 37 | 38 | 22 | 21 | 20 | 36 | 28 | 28 |
| P1 | 12 | 1 |  | 1 | 1 |  | 1 | 1 | 2 | 1 | 1 | 1* | 2 |  |  |  |  |  |
| P2 | 15 | 2 |  | 2 |  | 2 | 2 | 2 | 1 |  |  |  | 2 |  |  |  |  |  |
| P3 | 15 | 2 |  | 2* |  | 2 | 1 | 2* | 2 | 1 | 2 |  | 2 | 2* | 1* |  |  |  |
| P4 | 8 | 2 |  |  |  |  |  | 1 | 1 |  | 2 |  |  | * |  |  |  |  |
| P5 | 15 | 2 |  |  |  |  |  | 2 | 2 | 1 | 1 |  |  |  |  |  |  |  |
| P6 | 7 | 1 |  |  |  |  | 1 | 2 | 1 | 1 | 1 |  |  |  |  |  |  |  |
| P7 | 14 | 2 |  |  |  | 2 | 1 | 2 | 1 | 1* | 1 |  | 2 | 1* |  |  |  |  |
| P8 | 19 | 2 |  | 1 |  |  |  | 2 | 2 | 2 | 2 |  | 2 | 1 |  |  |  |  |
| P9 | 4 | 1 |  |  |  |  |  | 1 |  |  | 1 |  | 1 | 1 |  |  |  |  |
| P10 | 9 |  |  |  |  |  | 1 | 2 |  |  |  |  | 2 |  |  |  |  |  |

MRI scans were manually traced onto Damasio templates. Lesion size** was calculated as % template damaged. For areas not comprehensively characterized by Damasio templates, analyses were combined with manual analysis of the structural scan with the help of a trained radiographer. As a consequence of this additional analysis, in some cases* lesion characterization does not overlap with data already published. Quantification of lesion: 2 = complete destruction/serious damage to cortical grey matter; 1 = partial destruction/mild damage to cortical grey matter; empty = intact. Anatomical abbreviations: SMA/PMC: of Supplementary Motor Area/ Premotor Cortex; FP: Frontal Pole; DLPFC: Dorsolateral Prefrontal Cortex; ant-IFG: Inferior Frontal Gyrus, pars orbitalis; mid-IFG: Inferior Frontal Gyrus, pars triangularis; post-IFG: Inferior Frontal Gyrus, pars opercularis; SMG: Supramarginal Gyrus; AnG: Angular Gyrus; pMTG: posterior Middle Temporal Gyrus; STG: Superior Temporal Gyrus; MTG: Middle Temporal Gyrus; ITG: Inferior Temporal Gyrus; FuG: Fusiform Gyrus; TP: Temporal Pole; PHG: Parahippocampal Gyrus; Hpc: Hippocampus.

Supplementary Table 3. Semantic background tests

| Test | Max | Control Mean (SD) | Patient Mean | P1 | P2 | P3 | P4 | P5 | P6 | P7 | P8 | P9 | P10 |
| --- | --- | --- | --- | --- | --- | --- | --- | --- | --- | --- | --- | --- | --- |
| *Cambridge Semantic Battery* | |  |  |  |  |  |  |  |  |  |  |  |  |
| Picture Naming | 64 | 59a | 40.5 | **1** | 61 | **19** | **50** | 60 | **50** | **3** | **43** | **56** | 62 |
| Word-Picture Matching | 64 | 62.7a | 61.4 | 63 | 62 | **60** | 64 | **62** | **62** | **52** | 63 | 64 | **62** |
| Word CCT | 64 | 60.7 (2.06) | 50.1 | **39** | **43** | **29** | **53** | 59 | **52** | 57 | **48** | 61 | 60 |
| Picture CCT | 64 | 58.9 (3.1) | 49.7 | **31** | **44** | **45** | 56 | **45** | 57 | 54 | **51** | 53 | 61 |
| *Ambiguity task* | |  |  |  |  |  |  |  |  |  |  |  |  |
| Miscued dominant | 30 | 30 (0) | 18.9 | **12** | **13** | **13** | **14** | **20** | **19** | **21** | **27** | **24** | **26** |
| Miscued subordinate | 30 | 29 (1.20) | 13.5 | **7** | **10** | **14** | **8** | **10** | **15** | **18** | **16** | **18** | **19** |
| No cue dominant | 30 | 29.5 (0.54) | 24.4 | **22** | **18** | **24** | **22** | **24** | **26** | **27** | **25** | **28** | **28** |
| No cue subordinate | 30 | 28.9(0.64) | 16.1 | **11** | **9** | **14** | **14** | **19** | **17** | **19** | **18** | **21** | **19** |
| Cued dominant | 30 | 30 (0) | 23.8 | **23** | **21** | **19** | **22** | **24** | **23** | **23** | **27** | **27** | **29** |
| Cued subordinate | 30 | 29.75 (0.46) | 22.1 | **25** | **14** | **20** | **18** | **19** | **28** | **24** | **25** | **23** | **25** |
| *Synonym with distractors* | |  |  |  |  |  |  |  |  |  |  |  |  |
| Strong | 42 | 39.87 (2.23) | 19.2 | **15** | **12** | **13** | **20** | **21** | **23** | **30** | NT | **22** | **17** |
| Weak | 42 | 41.50 (0.53) | 28.4 | **25** | **23** | **29** | **24** | **27** | **30** | **31** | NT | **28** | **39** |
| *Object use* |  |  |  |  |  |  |  |  |  |  |  |  |  |
| Alternative | 37 | 33.67a | 22.2 | **14** | **13** | **14** | **21** | 34 | **22** | **22** | **27** | **26** | **29** |
| Canonical | 37 | 35.9 a | 34.3 | **32** | **31** | **29** | **35** | 37 | **35** | **33** | 37 | 37 | 37 |

Legend: Scores are number of correct; a = normal cut-off, NT = unavailable for testing, Bold underlined numbers denotes impaired scores (less than two standard deviation below mean).

Supplementary Table 4. Non-semantic background tests

| Test | Max | Controls Mean (SD) | Patients  Mean | P1 | P2 | P3 | P4 | P5 | P6 | P7 | P8 | P9 | P10 |
| --- | --- | --- | --- | --- | --- | --- | --- | --- | --- | --- | --- | --- | --- |
| *Non-semantic language tests* | | | | | | | | | | | | | |
| PALPA 9 real word repetition (total) | 80 | 73a | 60 | NA | **71** | **42** | 75 | 78 | 79 | **1** | NT | 74 | 77 |
| Category Fluency (8) | - | 62a | 27.8 | NA | 26 | **15** | **26** | **14** | **26** | NA | **4** | 80 | **57** |
| Letter Fluency (F, A, S) | - | 18a | 6 | NA | 2 | **2** | **6** | **3** | **6** | NA | **3** | **16** | **9** |
| Cookie theft (words/minute) | - | - | 24.85 | 0 | 18 | 9 | 12 | 60 | 37 | 0 | 21.5 | 54 | 37 |
| *Executive and spatial processing* | | | | | | | | | | | | | |
| TEA: counting without distraction | 7 | 4.2a | 5.14 | **4** | 5 | 6 | 5 | **4** | NT | 7 | 7 | 5 | 7 |
| TEA: counting with distraction | 10 | 2.6a | 2 | **2** | 3 | **1** | **1** | **2** | NT | 7 | 3 | **2** | 6 |
| Raven's coloured matrices (total) | 36 | 32.9 (2.41) | 28.3 | 31 | 29 | 31 | **24** | **19** | 30 | 34 | 31 | **21** | 33 |
| Brixton spatial anticipation (correct) | 54 | 28a | 23.44 | **21** | **7** | **18** | **26** | **24** | **23** | 31 | NT | 31 | 30 |
| Trial Making Test A (correct) | 24 | 24a | 23.1 | **19** | **22** | **23** | 24 | 24 | **23** | 24 | 24 | 24 | 24 |
| Trial Making Test B (correct) | 23 | 17a | 14.4 | **2** | 23 | **16** | **12** | **1** | **5** | 23 | 21 | 19 | 22 |
| *Visuospatial processing* | | | | | | | | | | | | | |
| VOSP dot counting | 10 | 8a | 9.33 | **7** | 10 | 10 | 9 | 10 | 10 | 8 | NT | 10 | 10 |
| VOSP position discrimination | 20 | 18a | 17.56 | 19 | 20 | **4** | 19 | **17** | 20 | 19 | NT | 20 | 20 |
| VOSP number location | 10 | 7a | 8.88 | 8 | 10 | **5** | 10 | 10 | 10 | 10 | NT | **5** | 8 |
| VOSP cube analysis | 10 | 6a | 8.13 | 8 | 9 | **4** | **4** | 7 | 9 | 10 | NT | 10 | 8 |
| *Wechsler Memory Scale* | | | | | | | | | | | | | |
| Digit Span Forward | 9 | 6.82 (0.64) | 3.5 | **0*** | **5** | **4** | **2** | 6 | **4** | **2*** | **5** | **3** | **4** |
| Digit Span Backward | 8 | 5.6 (0.97) | 1.5 | **0*** | **2** | **2** | **0** | **2** | **0** | NA | **3** | NT | **3** |
| Spatial Span Forward | 19 | 10(3)b | 6.8 | 10 | 5 | 10 | 6 | 5 | **3** | 6 | 7 | 7 | 9 |
| Spatial Span Backward | 19 | 10(3)b | 7.1 | 8 | **2** | 10 | 6 | **3** | **3** | 9 | 10 | 10 | 10 |
| Face Recognition Immediate | 19 | 10(3)b | 10.7 | 9 | 10 | 12 | 14 | **17** | 13 | 10 | 7 | 9 | 6 |
| Face Recognition Delayed | 19 | 10(3)b | 12 | 8 | 13 | 11 | 11 | 18 | 13 | 15 | 13 | 8 | 10 |

Legend: Scores are number of correct. a= Normal cut-off; b=WMS Age adjusted scaled score (SD); Bold underlined numbers denotes impaired scores (less than two standard deviations below mean); NT = unavailable for testing; NA = testing was not attempted because patients were non-fluent; TEA = Test of Everyday Attention; VOSP = Visual Object and Space Processing battery. Digit Span: participants were required to immediately retrieve numbers sequences of increased length, in forwards or backwards order. * = For non-fluent patients we used a paper with numbers wrote down during recall. Patients were instructed to point to each number in the same sequence in which was vocally presented by the experimenter. The sheet was not available during the presentation of numbers to avoid the use of spatial strategy for retaining the sequence in working memory. Face Recognition: participants were asked to remember 24 unfamiliar faces, presented one at time. Memory was tested immediately and following a delay of 25-35 minutes (delayed condition); participants identified 24 target faces amongst 48 stimuli, responding either “yes” or “no” to each face.

Supplementary Table 5. Descriptive statistics Experiment 1, 2, 3 and 4: accuracy and error type

|  | Experiment 1 | | | Experiment 2 | | | | Experiment 3 | | Experiment 4* | |
| --- | --- | --- | --- | --- | --- | --- | --- | --- | --- | --- | --- |
|  | Patients | Controls | | Patients | | Controls | | Patients | Controls | Patients | Controls |
|  | M (SD) | M (SD) | | M (SD) | | M (SD) | | M (SD) | M (SD) | M (SD) | M (SD) |
| Accuracy % | | | | | | | | | | | |
| Correct | 45.6 (20.1) | 83.7 (13.3) | | 52.1 (15.3) | | 93.4 (6.6) | | 55 (18.3) | 88.7 (6.5) | 61.3 (11) | 90.4 (3.9) |
| Related | 60.9 (15.5) | 87.7 (9.8) | | - | | - | | 54.1 (17.5) | 85.3 (11.4) | 64.6 (13.3) | 94.1 (5.0) |
| Unrelated | 30.3 (26.5) | 79.6 (17.6) | | - | | - | | 55.9 (21.2) | 92.1 (6.2) | 58 (8.9) | 86.7 (4.8) |
| Episodic strong/trained* | 47.2 (24.8) | 88.7 (10.9) | | 54.1 (16.7) | | 95 (6.5) | | 59.7 (19.7) | 93.3 (4.9) | 54 (13.8) | 90.9 (5.3) |
| Episodic weak/untrained* | 44.1 (17.3) | | 78.6 (16.5) | | 50 (17.3) | | 91.8 (7.7) | 50.3 (17.8) | 84.1 (9.8) | 68.6 (11.3) | 89.8 (4.2) |
| Errors % | | | | | | | | | | | |
| Related trials SEM/ | 37.3 (27.7) | 8.4 (13.5) | | - | | - | | 22.4 (13.7) | 10.5 (16) | 42.3 (12.6) | 38.7 (10.7) |
| Trained trials EPI* |  |  |  |  |  |  |  |  |  |  |  |
| Unrelated trials SEM/ | 27.9 (18.5) | 5.7 (10) | | - | | - | | 13.6 (18.8) | 27.4 (39.5) | 25.3 (6.9) | 46.5 (16) |
| Untrained trials UNR1* |  |  |  |  |  |  |  |  |  |  |  |
| Related trials SEMEP/ | 58.8 (30.3) | 76.5 (32) | | - | | - | | 61.9 (21.2) | 85.8 (20.9) | 11.4 (6.2) | 2.4 (5.6) |
| Trained trials FAM* |  |  |  |  |  |  |  |  |  |  |  |
| Unrelated trials SEMEP/ | 47.7 (16.2) | 30.9 (34.9) | | - | | - | | 43.9 (23) | 22.3 (30.9) | 9.4 (5.2) | 6.5 (10.2) |
| Untrained trials UNR2* |  |  |  |  |  |  |  |  |  |  |  |
| Related trials EP/ | 3.9 (5.9) | 7.4 (14.8) | | - | | - | | 15.7 (10.5) | 3.6 (8.9) | 5.6 (5.8) | 3.5 (5.9) |
| Trained trials UNR 3* |  |  |  |  |  |  |  |  |  |  |  |
| Unrelated trials EP/ | 24.4 (22.4) | 21.1 (28.1) | | - | | - | | 42.5 (28) | 42.6 (39.2) | 6 (3.4) | 2.4 (4.4) |
| Untrained trials UNR 3* |  |  |  |  |  |  |  |  |  |  |  |
| Proactive interferences | 40.7 (15.3) | 17.1 (12.9) | | 33.3 (14.5) | | 8.3 (9) | | 19.9 (18) | 9.6 (9.24) | - | - |
| Perseverations | 31.9 (27.1) | 3.8 (13.9) | | 19.3 (22.4) | | 7.7 (27.7) | | 14.6 (21.3) | 0 (0) | - | - |

Legend. Scores are % of correct. For Exp. 1, 2, 3: Related and Unrelated = probe paired with a semantically related/unrelated target at encoding; Strong = repeated 5 times at encoding; Weak = presented only once at encoding; SEM = novel and semantically related to the probe; SEM+EP = semantically related to the probe and target word for another probe; EP = target on a different trial but not semantically related to the probe. For Exp. 4: Related and Unrelated = EPI/UNR1 distractors semantically related or unrelated with target; Trained = probe episodically-associated with episodic distractor during episodic training; Untrained = probe not presented during episodic training; EPI = episodic distractor associated with the probe during episodic training; FAM = associated with a different probe during episodic training; UNR1, 2, 3 = novel unrelated distractors.

1. Supplementary analysis of confidence in Experiments 3 and 4

1.1. Experiment 3 (episodic memory for pictures): The final model [-2LL = 5150.26] revealed that confidence was predicted by accuracy [F(1, 1451) = 69.85, p < .001], group [F(1, 1012) = 358.67, p < .001], relatedness of response [F(1, 1448) = 30.63 , p < .001] and episodic strength [F(1, 1448) = 76.28, p < .001]. There was an interaction of episodic strength by group [F(1, 1448) = 9.67, p = .002] and a four-way interaction of these factors [F(1, 1448) = 6.38, p = .012]. The effect of episodic strength was larger in the control group [patients: b = .19, F(1, 630.01) = 15.83, p < .001; controls: b = .43, F(1, 819.27) = 68.20 p < .001]. We investigated the four-way interaction by examining the effect of accuracy and relatedness in strong and weak trials separately for both groups. During strong trials, confidence ratings were predicted by accuracy in both groups [patients: b = 0.33, F(1, 308) = 18.13, p <.001; controls: b = .26, F(1, 412) = 4.87, p = .028]; additionally patients’ confidence was higher for semantically-related responses [b = .11, F(1,308) = 5.33, p = .022] but this effect was not found for controls [b = -.05, F < 1]. During weak trials, confidence ratings were predicted by accuracy in both groups [patients: b = .38, F(1, 308) = 27.88, p = .001; controls: b = .48, F(1, 410) = 11.76, p = .001] and by the relatedness of the response in both groups [patients: b = .15, F (1,307) = 4.72, b = .030; controls: b = .34, F (1,408) = 5.87, p = .016; see Supplementary Figure 1]. For both strong and weak trials there was no relatedness by accuracy interaction in both groups [F ≤ 1.7; p ≥ .220].

1.2. Experiment 4 (semantic memory with episodic distractors): In Experiment 4, episodically-trained distractors were present on half the trials; therefore it was not possible to examine the effect of the main experimental manipulations and the presence of episodic distractors in a single analysis. Consequently, we ran two analyses. The first examined confidence ratings as a function of response accuracy, group, episodic training and semantic relatedness of the target and distractor. The final model [-2LL = 7993.43] included only main effects of accuracy and group. Controls were more confident than patients [b = - .59, F(1, 19.05) = 5.62, p = .028] and both groups were more confident during correct vs. incorrect trials [main effect of accuracy: b = .42, F(1, 2414) = 174.67, p < .001].

In the second model we explored confidence ratings as function of type of error. This model examined incorrect and trained trials only, looking at effects of error type (EPI, FAM vs. UNR3), group and target-distractor relatedness. The final model [-2LL = 1094.72] included the main effects of type of error and group. Controls were more confident than patients [main effect of group: b = - .74, F(1, 19.90) = 4.76, p = .041] and both groups were more confident when the episodic distractor was selected [main effect of error type: b = -.39, F (1, 309.19) = 16.41, p <.001, Supplementary Fig. 1B]. However, there was no interaction between these factors.


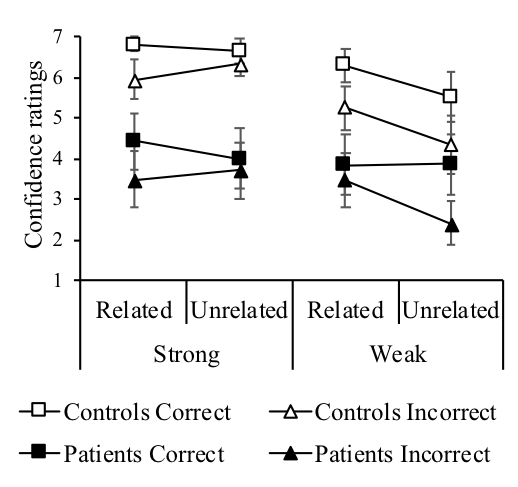


A)

B)


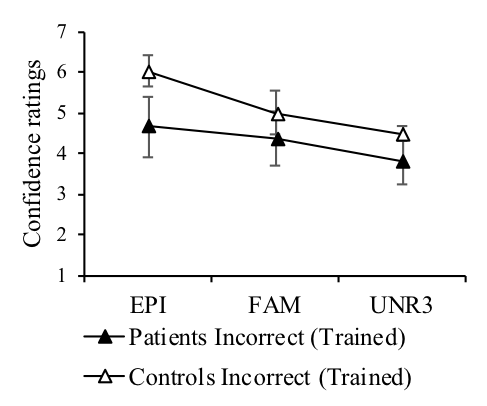


Supplementary Figure 1. Confidence ratings Experiment 3 (A) and 4 (B). Error bars show SE of mean.
